# Four-dimensional echocardiography and left ventricular systolic strain measured via two-dimensional speckle-tracking for Danon disease: a case series

**DOI:** 10.1093/ehjcr/ytab443

**Published:** 2021-11-16

**Authors:** Ma Changsheng, Jiali Fan, Zhou Bingyuan, Zhou Jiawei, Wang Li, Fan Lin, Liao Yuping, Zhao Caiming

**Affiliations:** The first Affiliated Hospital of Soochow University, Street Shizi 188, SuZhou, JiangSu Province, China

**Keywords:** 4D echocardiography, Case report, Danon disease, Left ventricular longitudinal strain

## Abstract

**Background:**

Danon disease is an X-linked multisystemic disorder characterized by skeletal myopathy, cardiomyopathy, and intellectual disability.

**Case summary:**

Herein, we describe two patients affected by Danon disease from the same family, a father (Patient 1) and his daughter (Patient 2). In Patient 1, a short PR interval with pre-excitation was evident. In Patient 2, over a 24-h period 2369 atrial premature beats and rare isolated ventricular ectopics were detected. Both patients exhibited left ventricular hypertrophy with non-compaction myocardium, and the left ventricular ejection fraction was impaired in Patient 1 and normal in Patient 2. In Patient 2, the total left ventricular strain value was reduced, and layer-specific strain revealed that subepicardial strain impaired more than in other layers. Late gadolinium enhancement was detected both in left and right ventricles in Patient 2, and cardiac fibrosis was more apparent in the subepicardium of left ventricular free wall. Four-dimensional (4D) echocardiography revealed that left atrial reservoir strain and left ventricular total longitudinal strain were induced.

**Discussion:**

Novel 4D echocardiography and left ventricular systolic strain may play important role in diagnosis and myocardial functional evaluation in Danon disease.

Learning points

- Non-compaction cardiomyopathy may be one of the two-dimensional (2D) echocardiographic characteristics present in Danon disease.
- Left ventricular strain measured via 2D speckling-tracking, especially for layer-specific strain, can be a more sensitive method for diagnosis and ventricular function evaluation in Danon disease.
- Four-dimensional volume and strain echocardiography may play important roles in the diagnosis and prognostic evaluation of Danon disease.

## Introduction

Danon disease is an X-linked multisystemic disorder that predominantly involves the mental retardation, skeletal myopathy, and hypertrophic cardiomyopathy.^1^ The gene mutation underlying Danon disease causes a deficiency of lysosome-associated membrane protein 2 (LAMP2).^2^ The dire consequences of LAMP2 mutations include in extensive replacement scarring, with autophagic vacuolated myocytes presumably containing degraded lysosomal material.^3^ Implantable cardioverter-defibrillators were evidently refractory to life-threatening ventricular tachyarrhythmias, and the only treatment intervention that effectively preserves life in Danon disease is heart transplantation.^4^ The aim of this case series was to highlight the use of four-dimensional (4D) echocardiography and left ventricular longitudinal strain, particularly for layer-specific strain in Danon disease.

## Timeline

**Table:** 

	Patient 1
1993	Suspected hypertrophic cardiomyopathy
June 2002	Echocardiography revealed a hypertrophic dilated left ventricle with poor function [left ventricular ejection fraction (LVEF) 24%].
2003	Died due to sudden cardiac arrest
	Patient 2
August 2011	Echocardiography and electrocardiography were normal
January 2019	Dyspnoea and palpitation while sleeping, echocardiography revealed left ventricular hypertrophy with preserved LVEF.
February 2019	Lysosome-associated membrane protein 2 mutation detected
December 2020	Left ventricular longitudinal strain and four-dimensional volume–strain performed

## Case presentation

### Patient 1

A 31-year-old man categorized as New York Heart Association Class IV was admitted to the hospital after suffering from anorexia for 2 weeks and dyspnoea for 1 week in August 2001. He had previously undergone surgery for appendicitis and had on other past medical history. He exhibited mild intellectual disability, and electrocardiography (ECG) depicted a short PR interval with pre-excitation and negative T waves (*Figure 1A*). Echocardiography revealed a hypertrophic dilated left ventricle with poor systolic function [left ventricular ejection fraction (LVEF) 24%] and signs suggestive of non-compaction myocardium (*Figure 1B*). Doppler parameters indicated reduced *e*′ velocity at the mitral valve annulus and increased tricuspid regurgitation velocity. A restrictive filling pattern was evident via pulse Doppler of the mitral valve. According to the American Society of Echocardiography/European Association of Cardiovascular Imaging guidelines and references,^5^ his left ventricular diastolic dysfunction was in Grade 3. There was severe mitral and tricuspid regurgitation. Abnormal laboratory parameters included elevated total bilirubin (85.3 µmol/L), indirect bilirubin (55.4 µmol/L), and aspartate aminotransferase (284 IU/L). He experienced sudden cardiac death at the age of 33 years.

**Figure 1:**
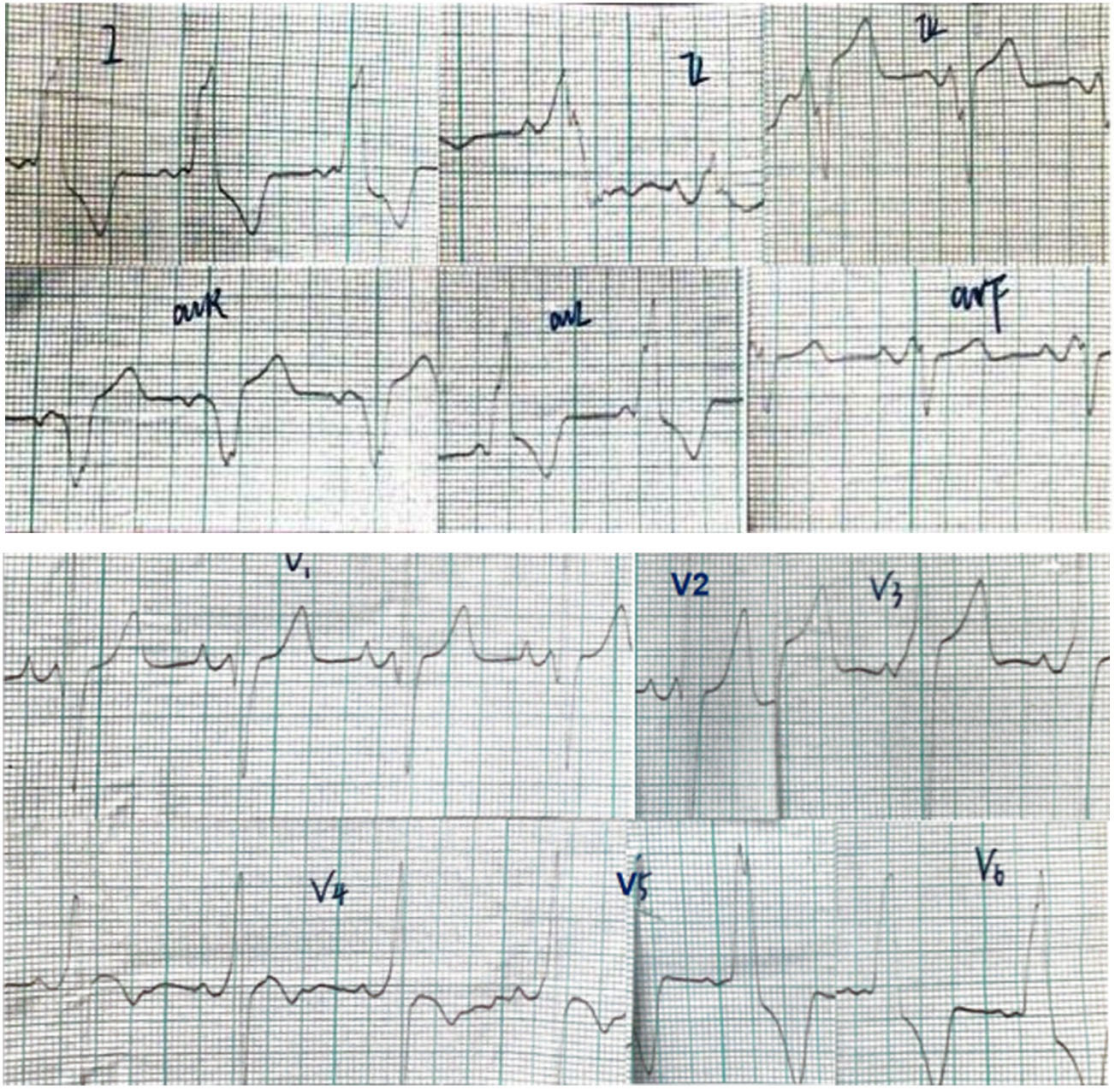
(A) electrocardiography of patient 1. (B) two-dimensional and Doppler echocardiography of patient 1.

**Figure 1:**
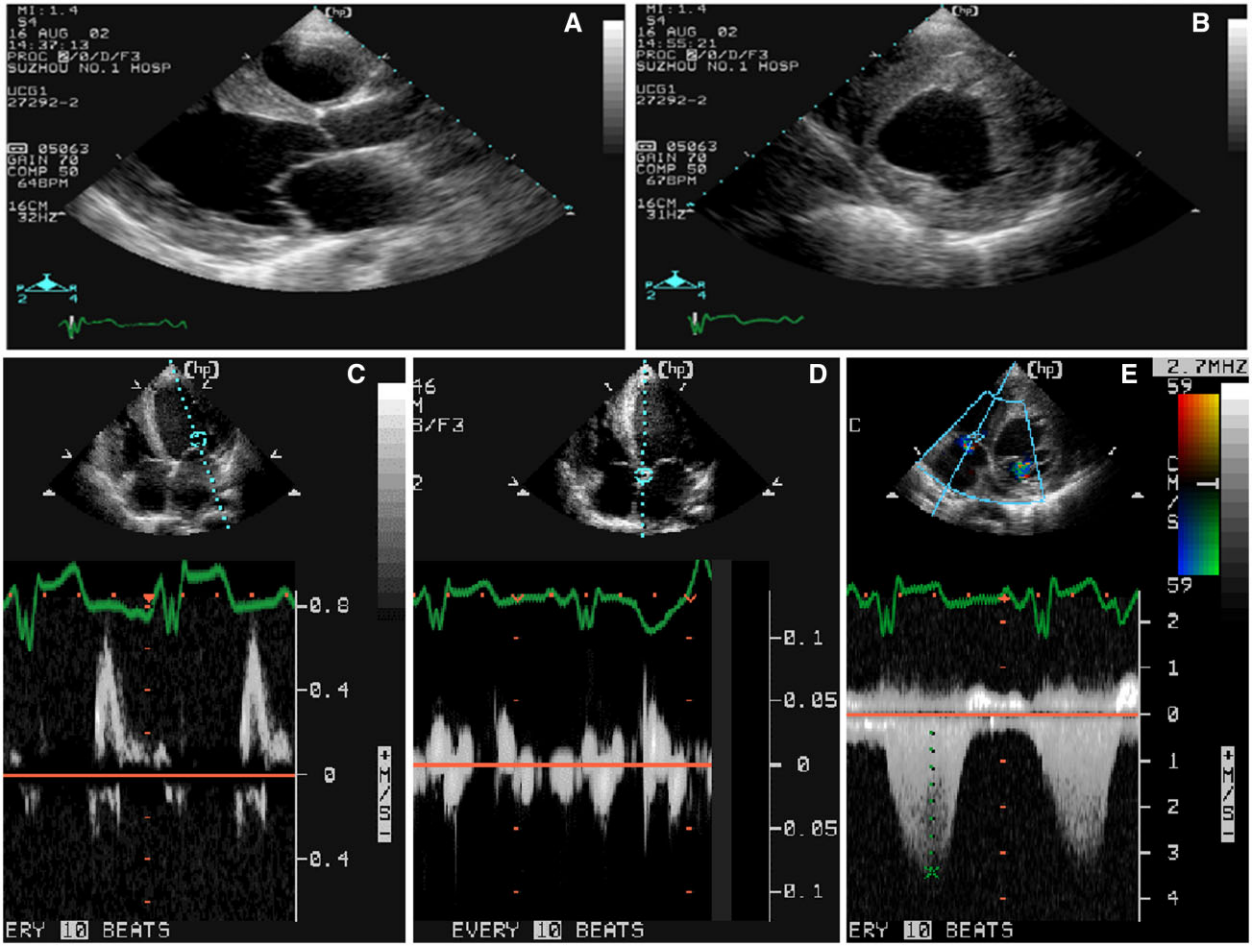
Continued.

### Patient 2

**Figure 2:**
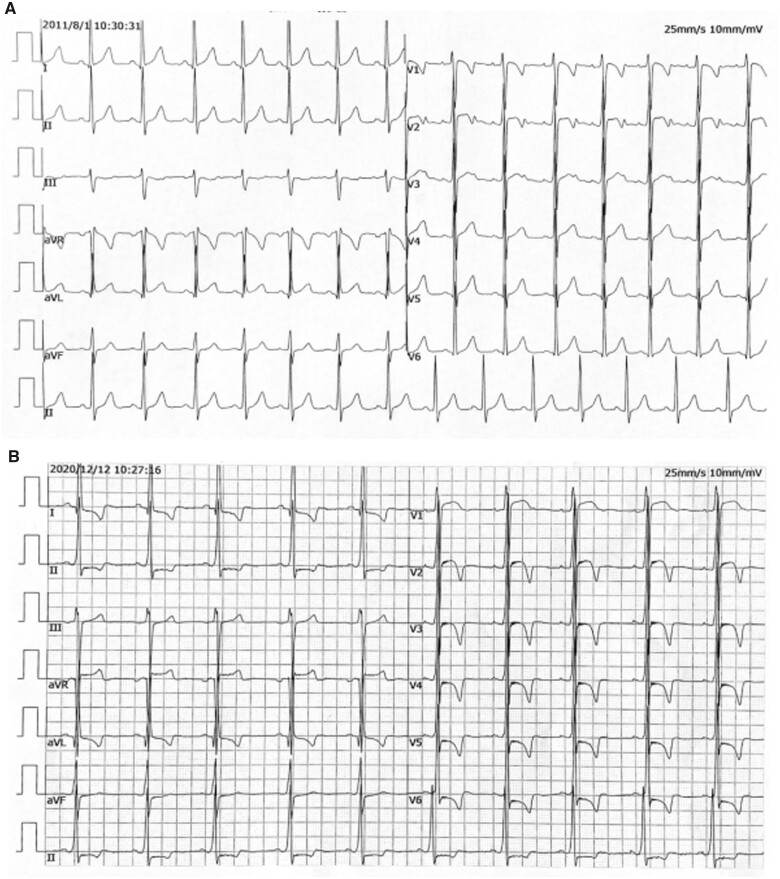
(A) Twelve-lead electrocardiography 10 years prior to patient 2’s current presentation. (B) Electrocardiography of patient 2 in December 2020.

Patient 1’s daughter (Patient 2) was admitted to hospital in February 2019 at the age of 19 years with dyspnoea and palpitation while sleeping for 6 months. She had no past medical history. Approximately 10 years prior, echocardiography and ECG had been performed and were normal (*Figure 2A*). She evidently had no learning difficulties. On physical examination, she appeared well developed and had normal skeletal muscle strength and muscle tone. Laboratory parameters included elevated aspartate aminotransferase (64 IU/L), creatine kinase-myocardial isoenzyme (7.5 ng/mL), lactate dehydrogenase (510 U/L), TnTI (0.09 ng/mL), N-terminal pro-brain natriuretic peptide (543.4 pg/mL), and lipoprotein(a) (0.93 g/L). Alanine aminotransferase (18 IU/L) and creatine kinase (108 U/L) were in normal ranges.

**Figure 3:**
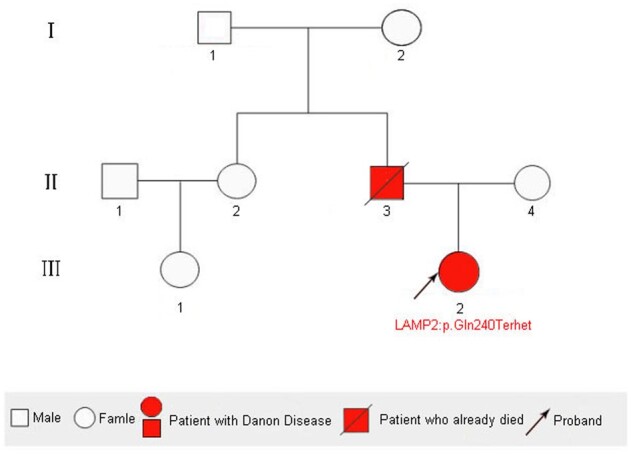
Pedigree chart of patient 1 and patient 2’s family.

Twelve-lead ECG indicated left ventricular hypertrophy with deep negative T waves, and the QRS duration was 120 ms (*Figure 2B*). In 24-h Holter monitoring, there were 2369 atrial premature beats (2.6% burden) and rare isolated ventricular ectopics. Coronary computed tomography angiography was normal. Echocardiography revealed left ventricular hypertrophy, and no significant left ventricular outflow tract obstruction was evident in resting echocardiography or dobutamine stress echocardiography (5–20 µg/kg/min). Blood was sent for genotyping and a heterozygous nonsense mutation in the LAMP2 gene (LAMP2: p.Gln240Ter het) was identified. She was diagnosed with Danon disease and started on an oral β-blocker followed by diltiazem administration. Her father (Patient 1) had died 17 years prior because of heart failure in the context of hypertrophic cardiomyopathy. A pedigree chart is shown in *Figure 3*. Three generations involving five family members were assessed, but no relevant mutations were detected in other family members.

**Figure 4:**
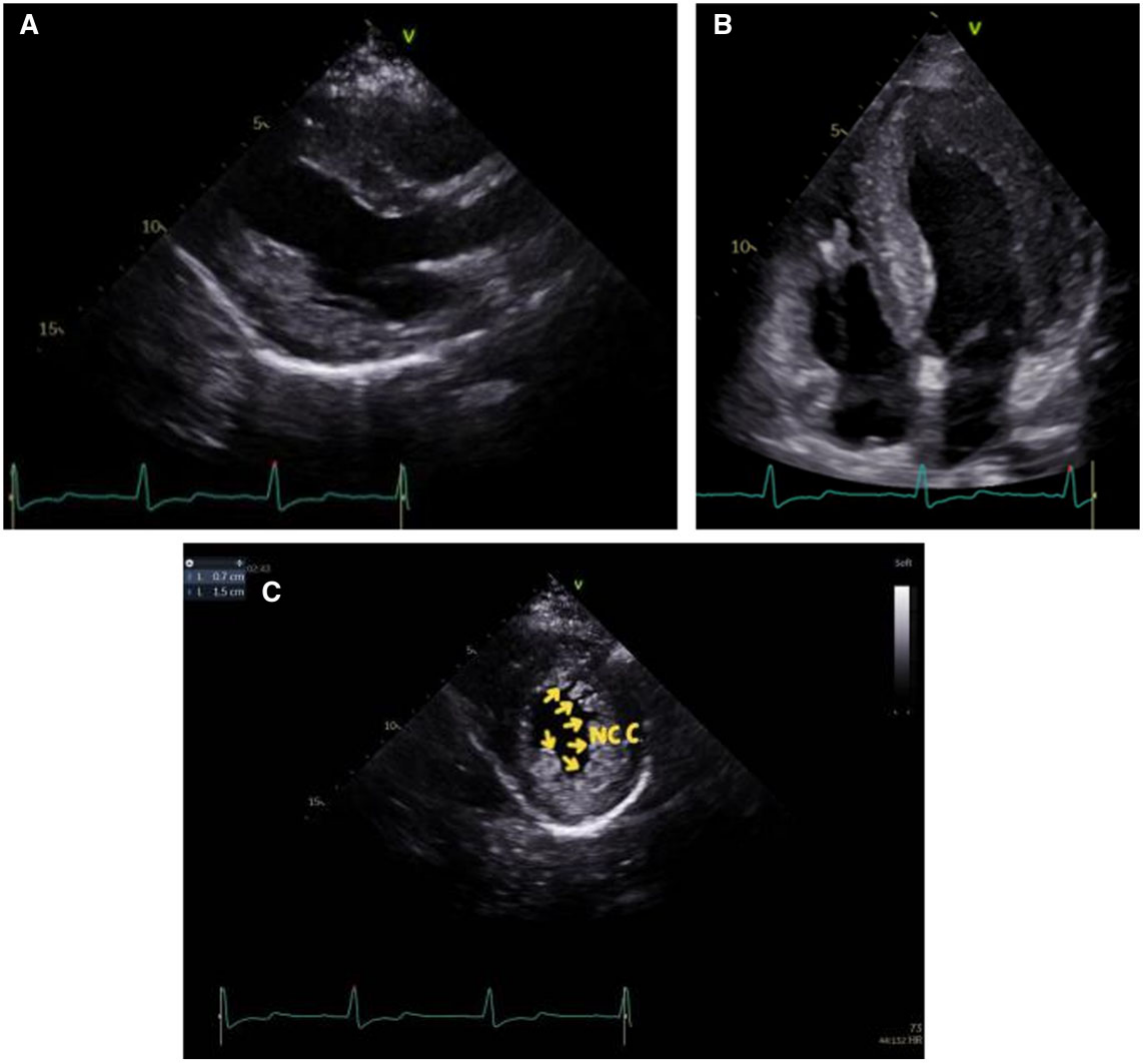
Two-dimensional echocardiography of patient 2. Arrows indicate non-compacted myocardium (NC). C indicates compacted myocardium.

In December 2020, echocardiography was performed again for Patient 2, including 4D echocardiography and two-dimensional (2D) left ventricular systolic strain. The 2D echocardiographic parameters were similar to the echocardiography performed 1 year ago, which revealed normal ventricular volumes and ejection fractions but demonstrated concentric left ventricular hypertrophy. Non-compaction myocardium characterized by prominent trabeculations and deep intertrabecular recesses was detected at the basal to apex ventricular segment of the left ventricular inferior, lateral, and anterior walls (*Figure 4*). The affected segments had a two-layer structure that consisted of a compact epicardial layer and an endocardial layer, and the endocardial layer that exhibited prominent trabecular meshwork and deep intertrabecular spaces. The ratio of non-compacted to compacted myocardial layers at the site of maximal wall thickness (22 mm) was 2.1. The LVEF measured via the biplane method was 65%, and the left atrial volume index was 22.1 mL/m^2^. Doppler parameters included septal *e*′ velocity 4 cm/s and lateral *e*′ velocity 6 cm/s, and *E*/*e*′ was 15.8, *E* wave velocity was 0.82 m/s, and *E*/*A* was 1.5, suggesting Grade 1 impaired diastolic dysfunction. The structure, function, and strain of the right ventricle were normal.

**Figure 5:**
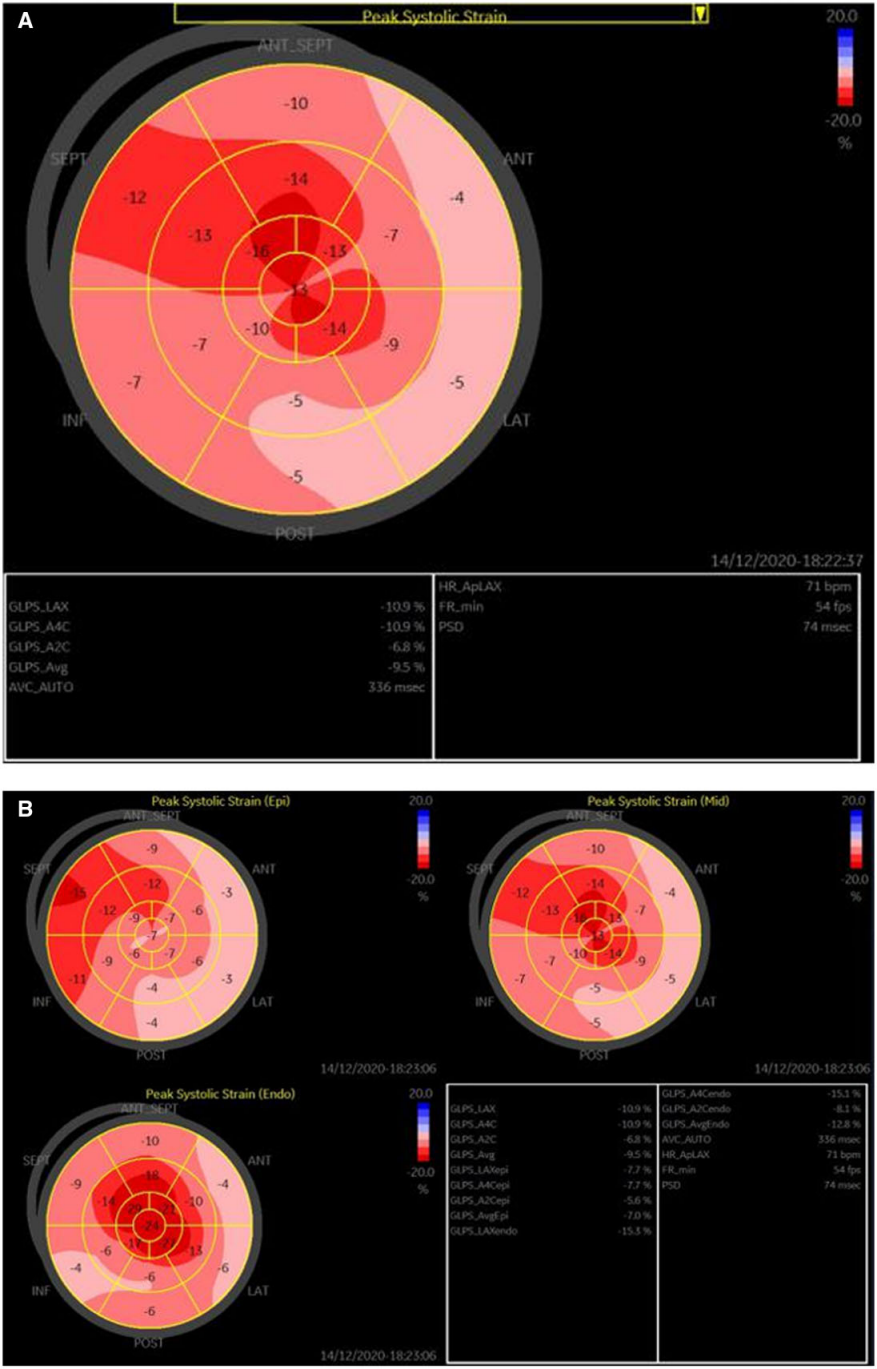
Left ventricular strain measured via speckle tracking. (A) The total left ventricular longitudinal strain decreased (-9.5%). (B) Layer-specific strain in patient 2 indicated that the reduction in myocardial strain was more apparent in the epicardium than in other layers.

Left ventricular strain (*Figure 5*) indicated global longitudinal strain decreased to −9.5%, which was less than it had been 1 year prior (−12.7%). Layer-specific strain indicated that the myocardial longitudinal peak strain of the epicardial layer reduced more than other layers, and the endocardial layer strain in the left ventricle apex was normal.

**Figure 6:**
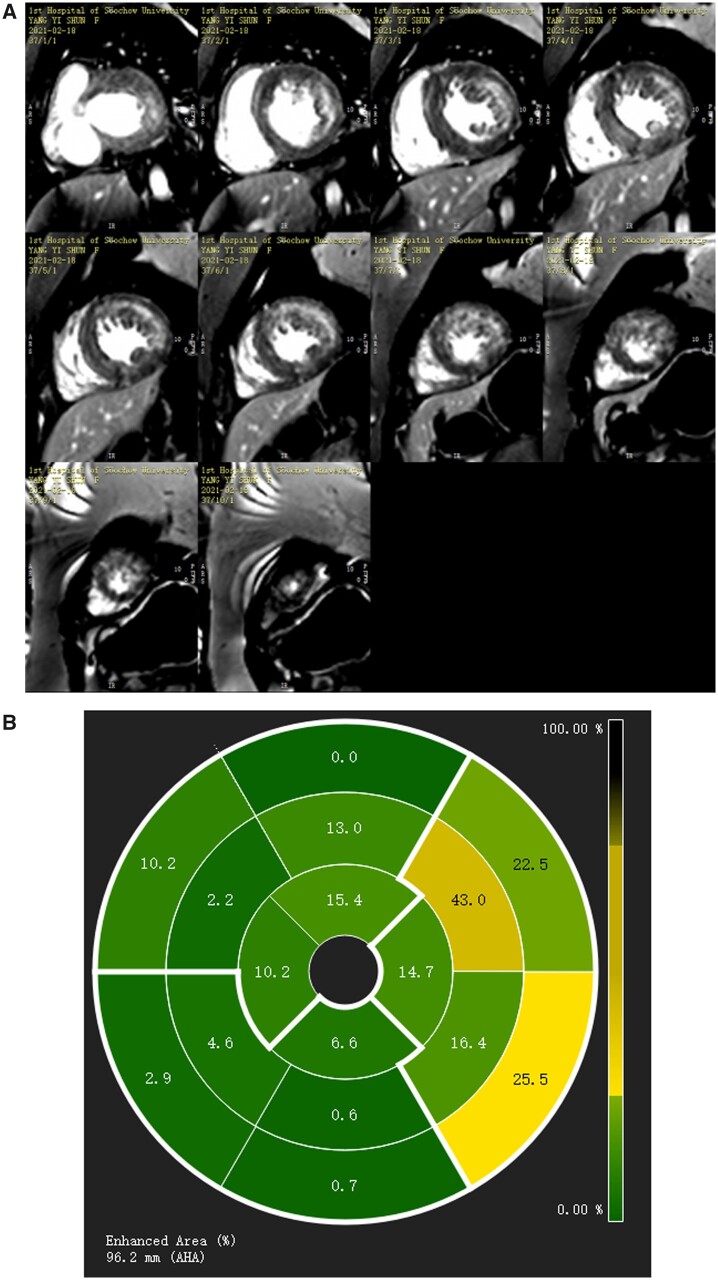
(A) Contrast-enhanced inversion recovery gradient echo image showing late gadolinium enhancement in both left and right ventricles. More cardiac scarring is detected in the epicardium of the left ventricular free wall. (B) Enhanced area percentage of late gadolinium enhancement.

*Figure 6* shows 4D echocardiography of Patient 2. Maximum left atrial volume was 31 mL, the left atrial ejection fraction was 57%, and the left atrial peak longitudinal strain reduced to 15%. Similar to 2D echocardiography, the left ventricular 4D volume and LVEF were normal, although the 4D left ventricular mass index was increased (119 g/m^2^) and left ventricular longitudinal strain was decreased (–6%).

**Figure 7:**
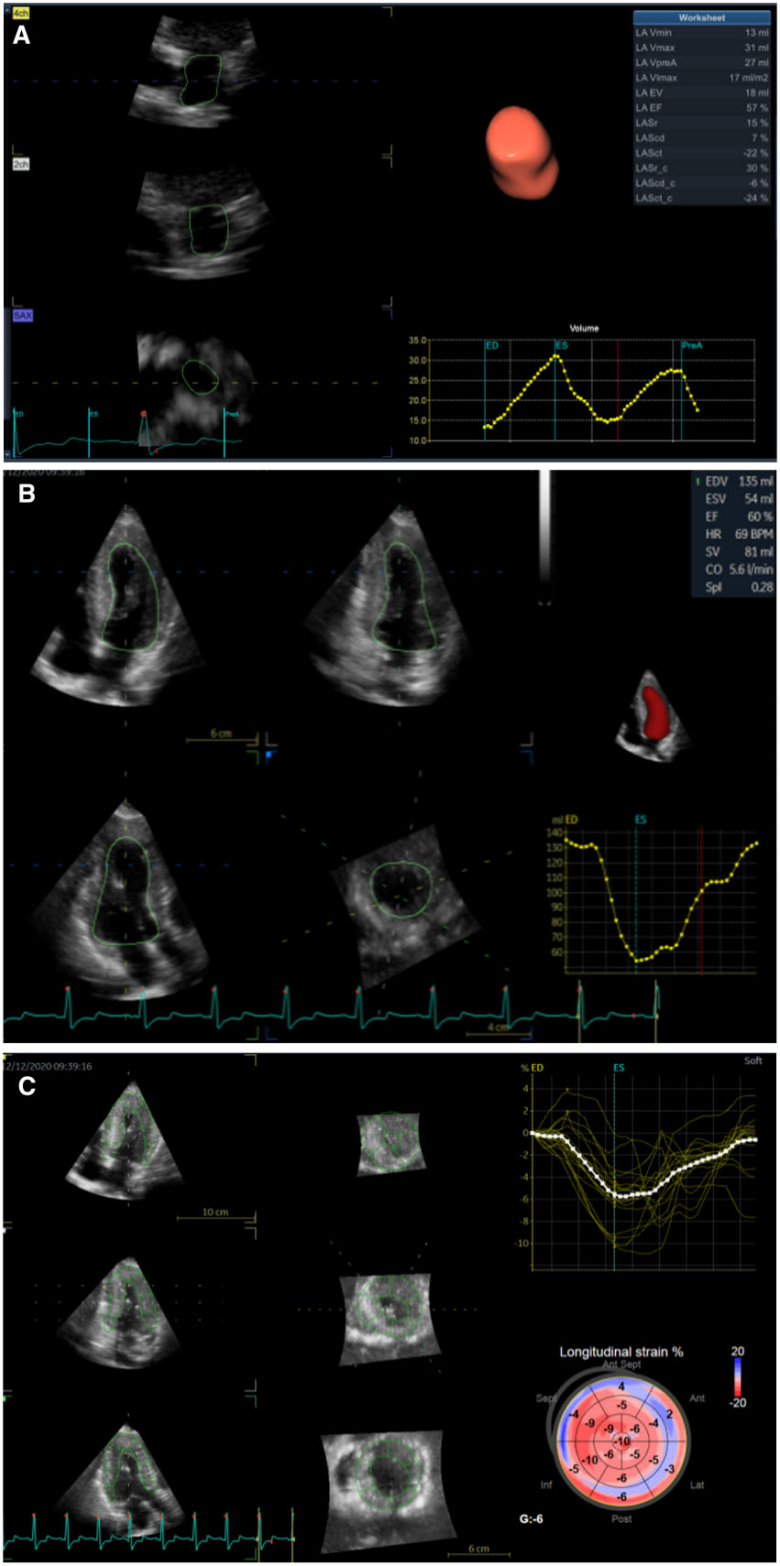
Four-dimensional echocardiography of patient 2. (A) Four-dimensional left atrial volume-strain echocardiography. (B) Four-dimensional left ventricular volume echocardiography. (C) Four-dimensional left ventricular strain.

Cardiac imaging depicted increased thickness of left ventricular wall, and late gadolinium enhancement (LGE) distributed in both right and left ventricular myocardium (*Figure 7*), and more LGE was evident in the epicardium of the free wall of the left ventricle. At the last follow-up (September 2021), she was clinically stable.

## Discussion

Danon disease has traditionally been defined as a clinical triad of skeletal myopathy, mental retardation, and cardiomyopathy. Both patients in the current series exhibited hypertrophic cardiomyopathy. The father exhibited mild intellectual disability whereas his daughter’s mental capacity was normal, which is concordant with previous clinical reports indicating that cognitive impairment evident in 70–100% of patients but in the majority of cases it is mild.^1^^,^^6^^,^^7^ It has been reported that skeletal myopathy is present in 50.7% of patients and usually affects the proximal muscles,^8^ and the disease process can also involve liver, retina, and lungs.^9^ No skeletal muscle weakness and other clinical abnormalities were detected in either of the present cases.

Cardiac electrical abnormalities are common in Danon disease patients, affecting 86–100% of males.^10^ The most frequent ECG abnormality is a pre-excitation pattern, which is present in 69% of males and 27% of females.^9^ In the current series, pre-excitation was evident on surface ECG in Patient 1. Recent evidence suggests that fasciculoventricular pathways, rather than atrioventricular pathway may be an alternate and dominant explanation for this in patients with Danon disease.^11^ Atrial and ventricular arrhythmias have also been described in Danon disease.^4^^,^^12^ In Patient 2 in the present series, there were 2369 atrial premature beats and rare isolated ventricular ectopics in 24 h, which is consistent with previous reports.

Two-dimensional echocardiography depicted left ventricular hypertrophy with non-compaction myocardium in both patients in the current series, and it was also reported by Tada *et al*.,^13^ suggesting that non-compaction myocardium can be one of the 2D echocardiographic characteristics observed in Danon disease. Danon disease is progressive, with preserved LVEF and normal cavity dimensions early in the course of disease.^1^ Echocardiography of Patient 2 was normal 10 years prior, but at the time of the current presentation, she exhibited hypertrophic left ventricle with diastolic dysfunction, suggesting that her cardiac function may deteriorate in the future.

Speckle-tracking echocardiography has recently enabled the measurement of myocardial strain and strain rate, which are evidently useful for the evaluation of cardiac function in Danon disease.^14^ In Patient 2 in the current series, LVEF was normal but the total left ventricular longitudinal strain had decreased, apparently less than 1 year prior, suggesting that myocardial function was substantially impaired. As well as conventional left ventricular strain, layer-specific strain was also performed in Patient 2, and it indicated that epicardial layer strain was impaired more than in other layers. Lysosome-associated membrane protein 2 mutations result in extensive replacement of scarring in left ventricular myocardium with autophagic and vacuolated myocytes presumably containing degraded lysosomal material. We suspect that these abnormalities infiltrated myocardium and converted epicardium to endocardium, which may account for the substantial decrease in the epicardial layer in left ventricular layer-specific strain. Cardiac fibrosis has also been confirmed in Danon disease via the use of cardiovascular magnetic resonance to quantify LGE in the previous reports.^15^ In Patient 2, LGE was distributed in both left and right ventricular myocardium, and cardiac fibrosis was more apparent in the epicardium of left ventricular free wall (*Figure 6*). The LGE results were consistent with the layer-specific strain, which suggested that the epicardial layer of the left ventricle was affected more than other layers.

Four-dimensional volume–strain echocardiography is a recently developed technique that can facilitate the acquisition of higher temporal resolution and spatial resolution with a higher frame rate, and it is less prone to artefacts than other methods. In Patient 2 in the present case series, 4D volume and strain echocardiography enable the evaluation of cardiac function and myocardial tissue characterization precisely and non-invasively in the current case series (*Figure 7*).

In conclusion, non-compaction myocardium was detected in both cases of the current case series, it may be one of 2D echocardiographic characteristics in Danon disease. Left ventricular strain evaluated left ventricular function more sensitively than LVEF. Layer-specific strain shows more significant impairment in the epicardium, which tallies nicely with the location and degree of LGE in the subepicardium. Novel 4D echocardiography and left ventricular systolic strain may play an important role in diagnosis and myocardial functional evaluation in Danon disease.

## Lead author biography

**Figure:**
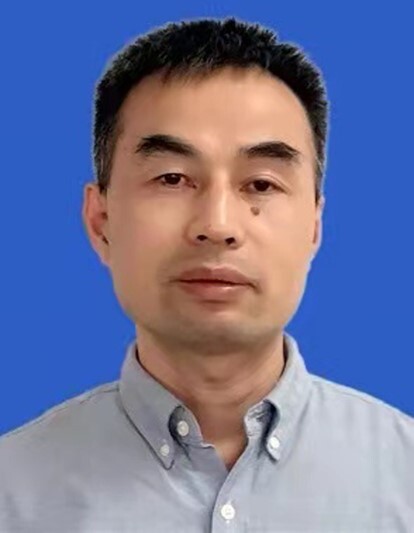


Zhou Bingyuan is a PHD of cardiovascular medicine.

## Supplementary material

Supplementary material is available at *European Heart Journal—Case Reports* online.

**Slide sets:** A fully edited slide set detailing these cases and suitable for local presentation is available online as Supplementary data.

**Consent:** The authors confirm that written consent for submission and publication of this case report including the images and associated text it contains has been obtained from patient in accordance with COPE guidelines.

**Conflict of interest**: None declared.

**Funding:** None declared.

## Supplementary Material

ytab443_Supplementary_Data
