# Supplementary material for: Four-dimensional echocardiography and left ventricular systolic strain measured via two-dimensional speckle-tracking for Danon disease: a case series
Source: Eur Heart J Case Rep. 2021 Nov 16;5(12):ytab443. doi: 10.1093/ehjcr/ytab443 (PMC8684807; doi:10.1093/ehjcr/ytab443)
Supplement: ytab443_Supplementary_Data [file ytab443_supplementary_data.pptx]

## Slide 1
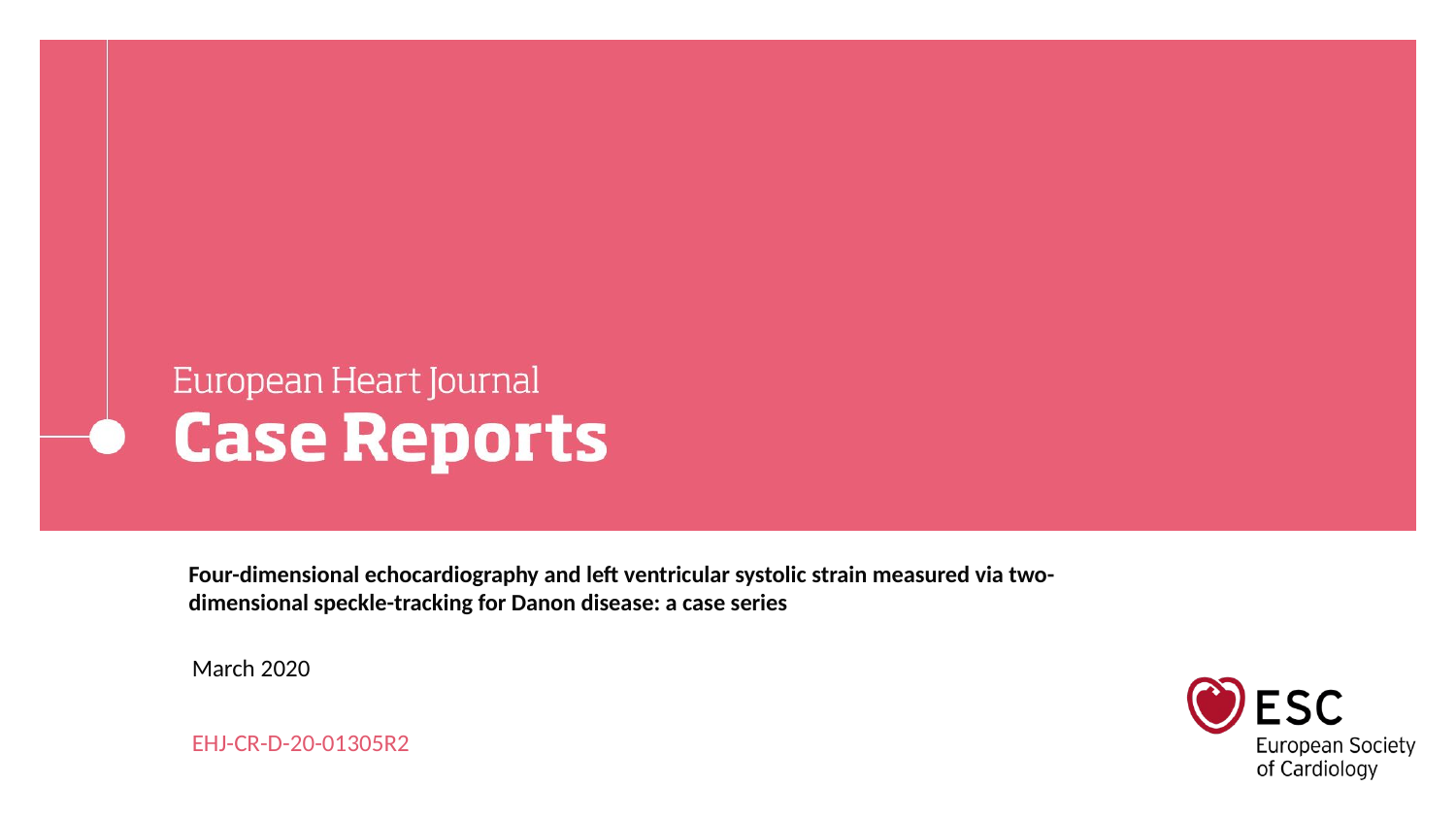

# Four-dimensional echocardiography and left ventricular systolic strain measured via two-dimensional speckle-tracking for Danon disease: a case series
March 2020
EHJ-CR-D-20-01305R2

## Slide 2
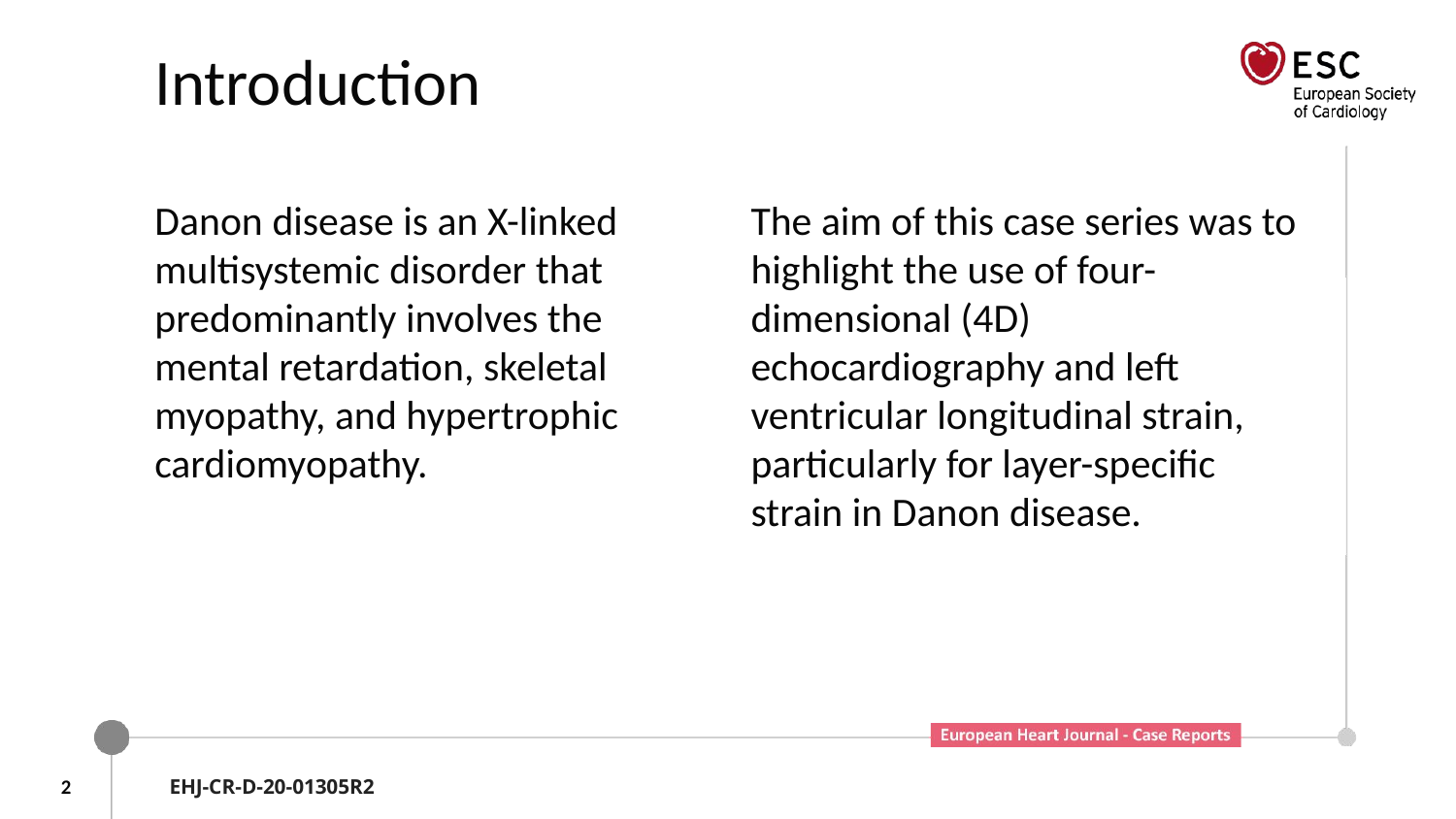

# Introduction
Danon disease is an X-linked multisystemic disorder that predominantly involves the mental retardation, skeletal myopathy, and hypertrophic cardiomyopathy.
The aim of this case series was to highlight the use of four-dimensional (4D) echocardiography and left ventricular longitudinal strain, particularly for layer-specific strain in Danon disease.
2
EHJ-CR-D-20-01305R2

## Slide 3
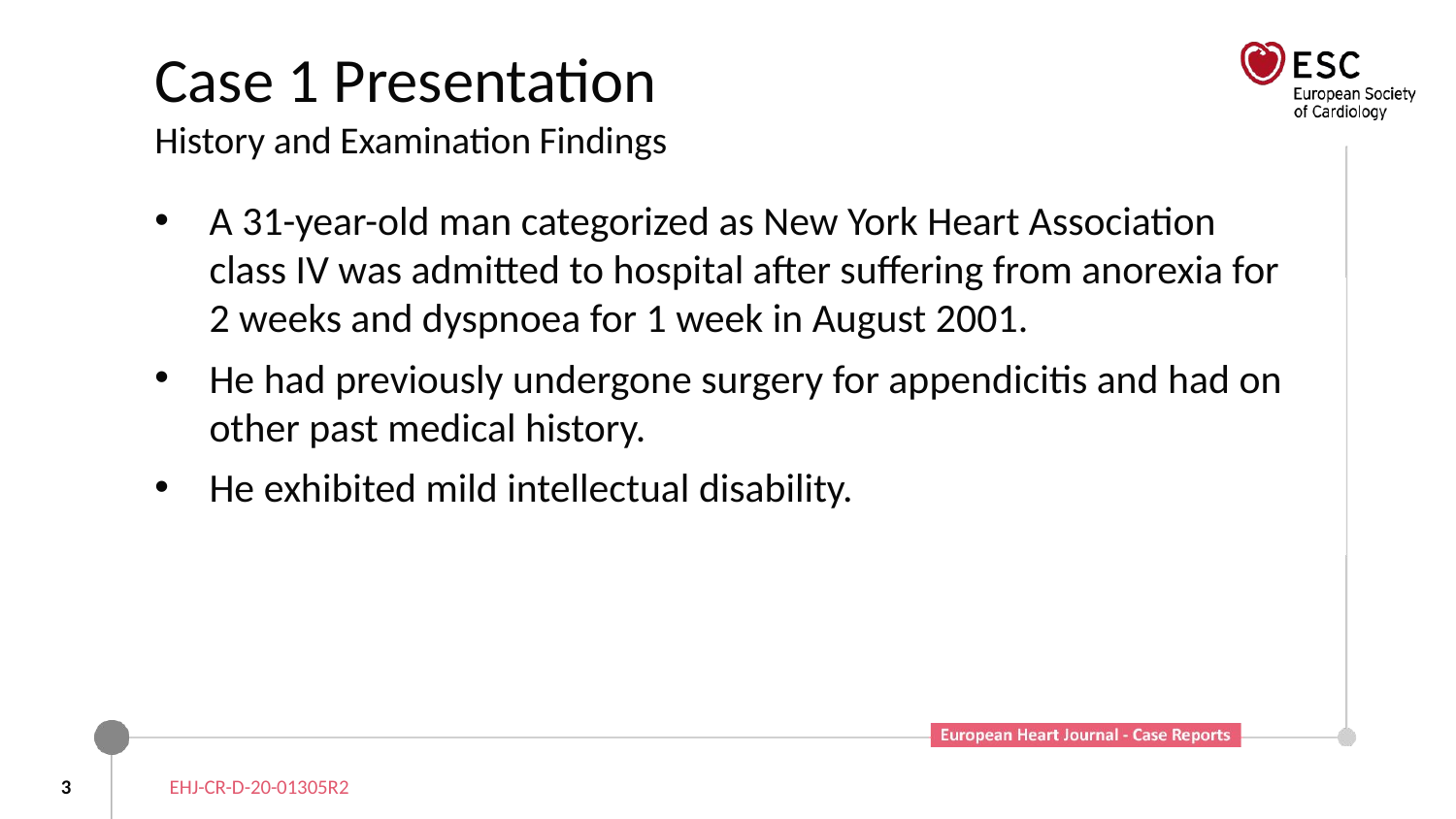

# Case 1 Presentation History and Examination Findings
A 31-year-old man categorized as New York Heart Association class IV was admitted to hospital after suffering from anorexia for 2 weeks and dyspnoea for 1 week in August 2001.
He had previously undergone surgery for appendicitis and had on other past medical history.
He exhibited mild intellectual disability.
3
EHJ-CR-D-20-01305R2

## Slide 4
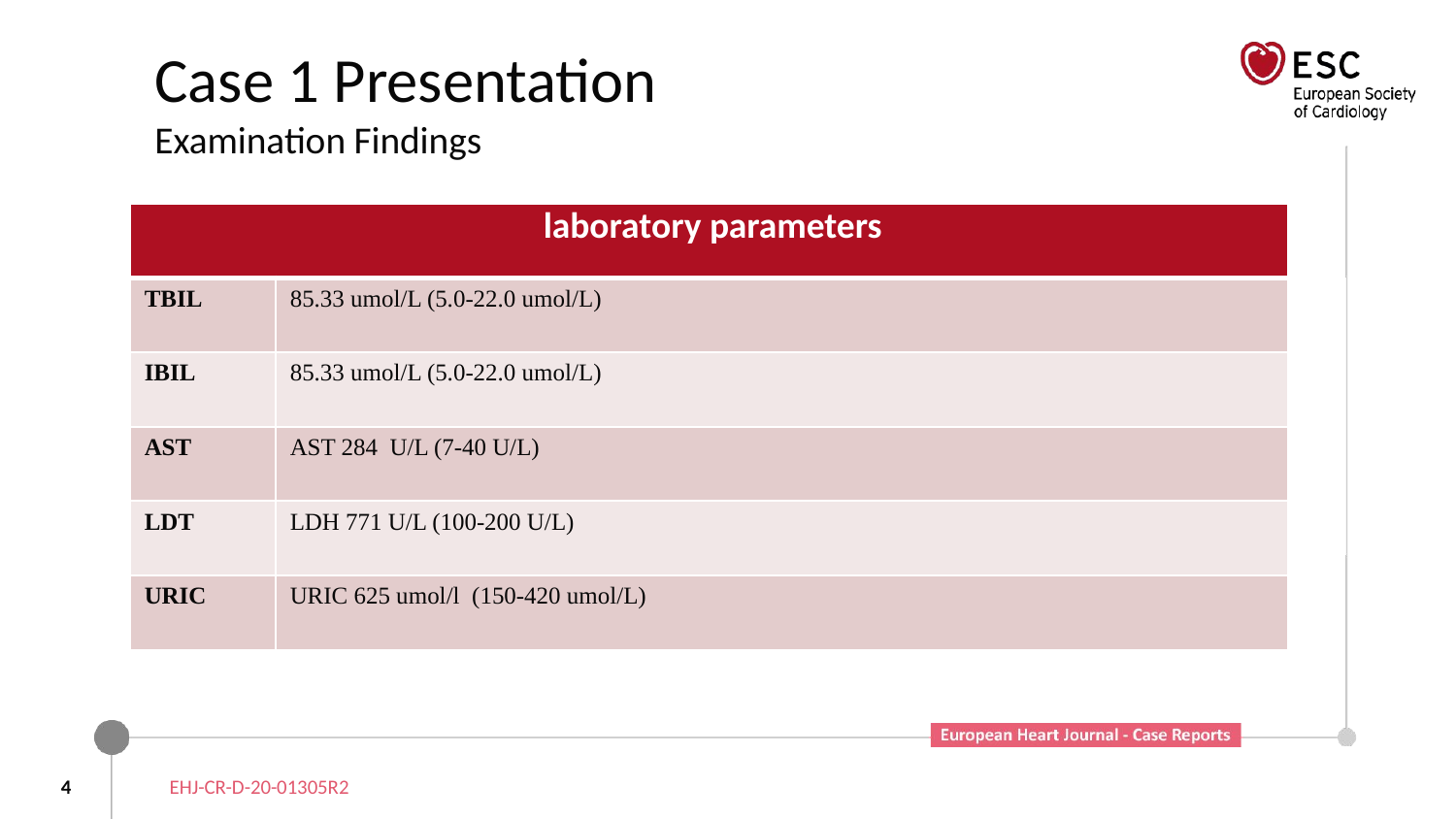

# Case 1 Presentation Examination Findings
| laboratory parameters | |
| --- | --- |
| TBIL | 85.33 umol/L (5.0-22.0 umol/L) |
| IBIL | 85.33 umol/L (5.0-22.0 umol/L) |
| AST | AST 284 U/L (7-40 U/L) |
| LDT | LDH 771 U/L (100-200 U/L) |
| URIC | URIC 625 umol/l (150-420 umol/L) |
4
EHJ-CR-D-20-01305R2

## Slide 5
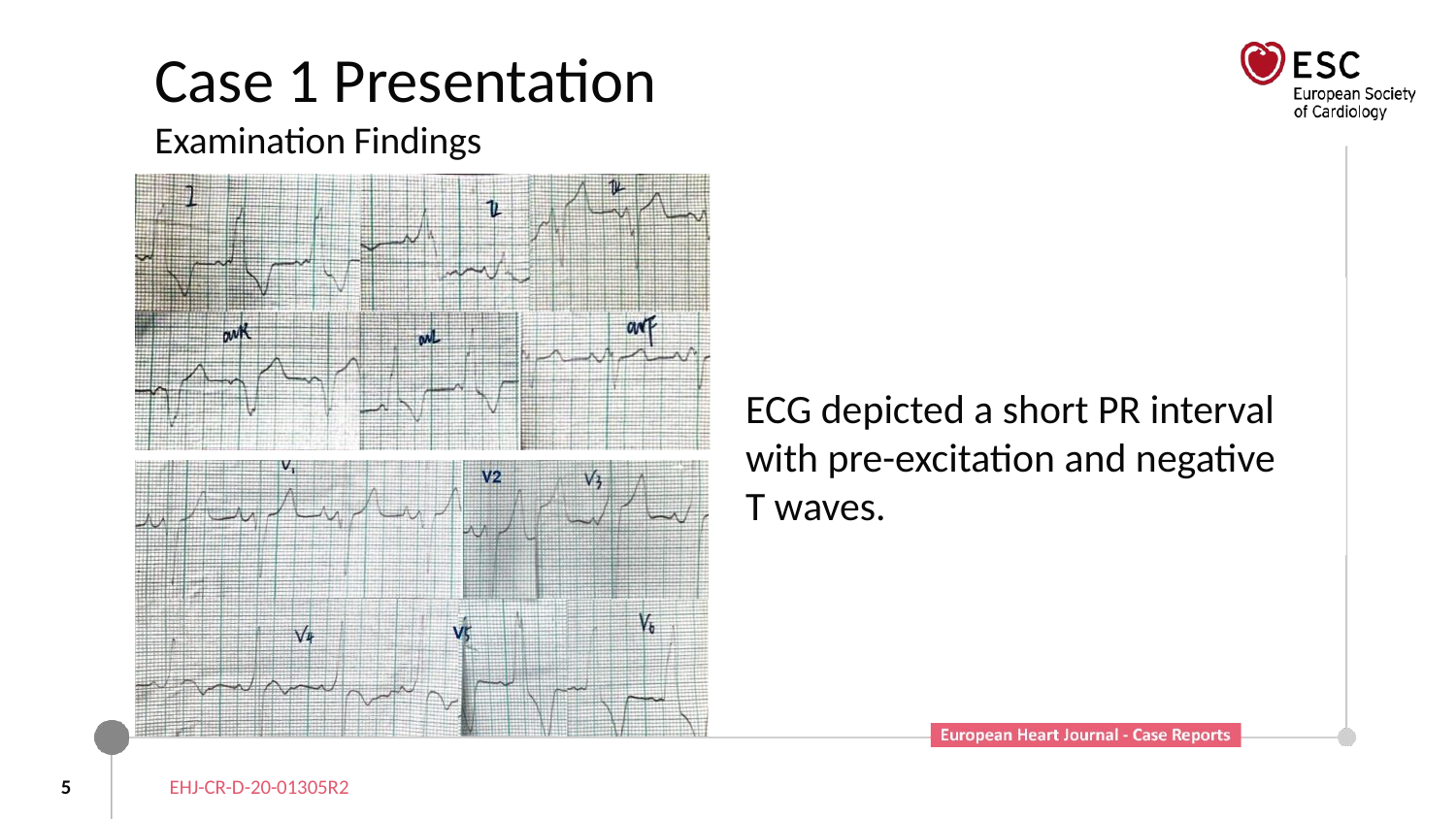

# Case 1 Presentation Examination Findings
ECG depicted a short PR interval with pre-excitation and negative T waves.
5
EHJ-CR-D-20-01305R2

## Slide 6
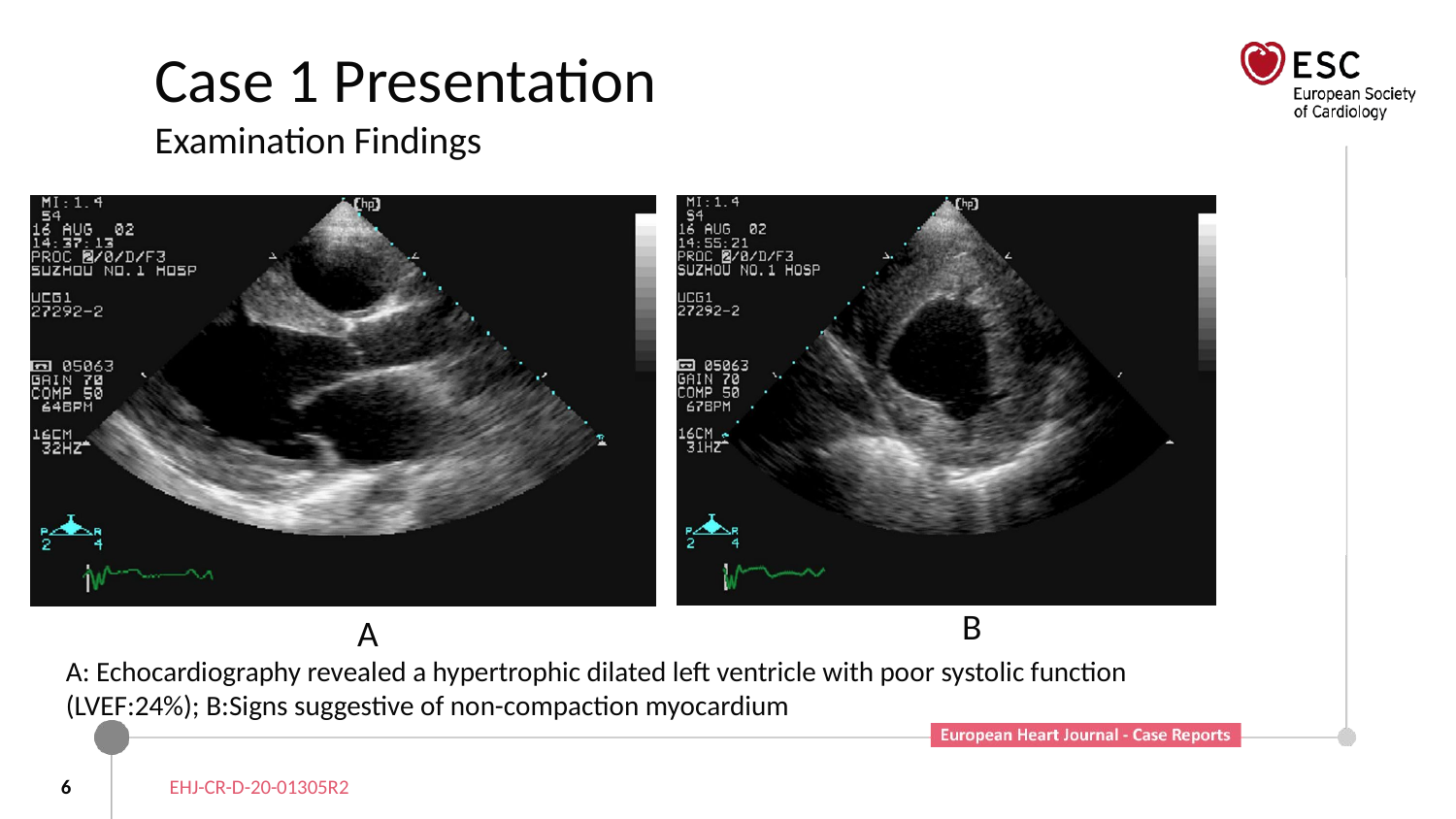

# Case 1 Presentation Examination Findings
B
A
A: Echocardiography revealed a hypertrophic dilated left ventricle with poor systolic function (LVEF:24%); B:Signs suggestive of non-compaction myocardium
6
EHJ-CR-D-20-01305R2

## Slide 7
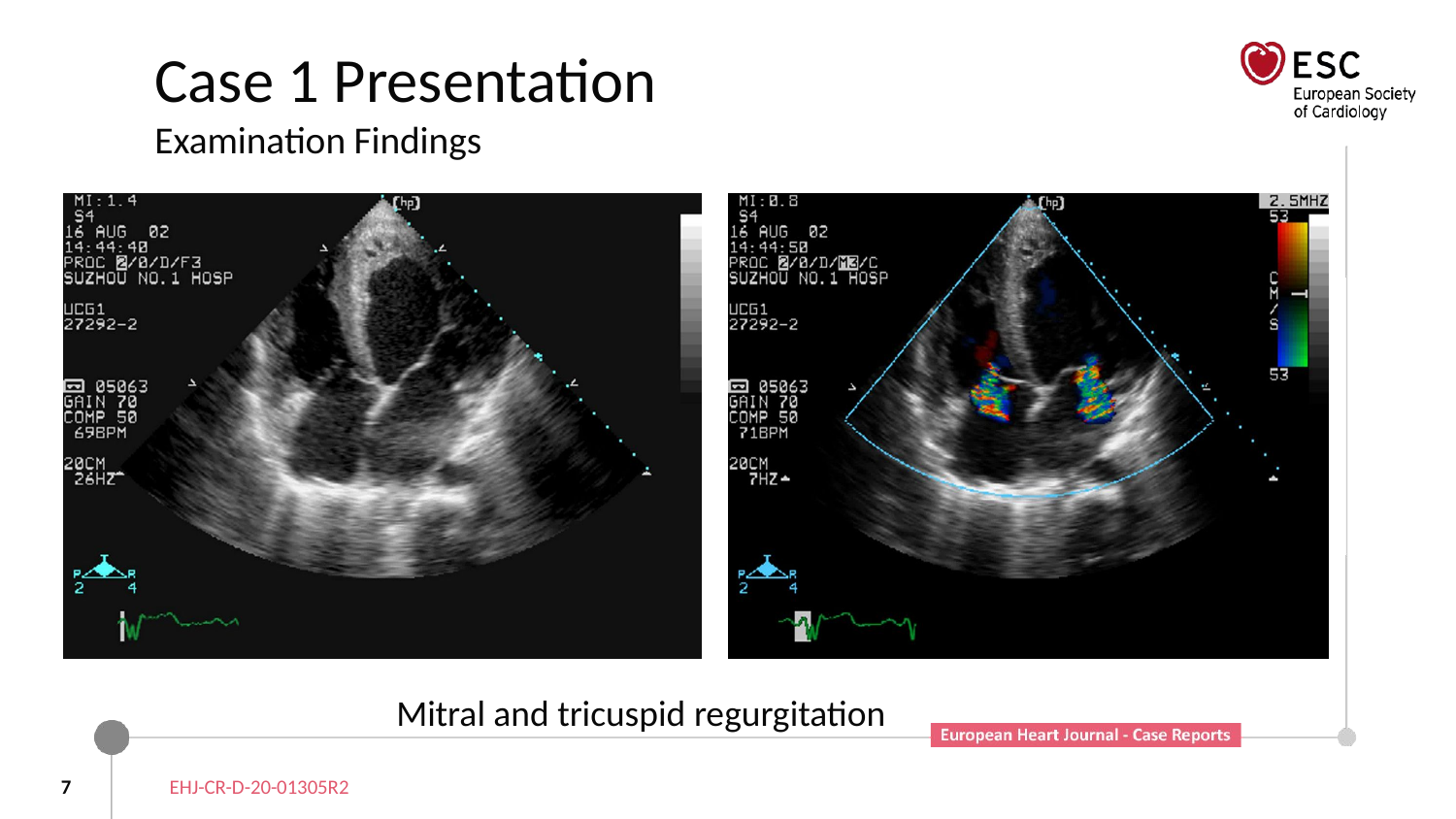

# Case 1 Presentation Examination Findings
Mitral and tricuspid regurgitation
7
EHJ-CR-D-20-01305R2

## Slide 8
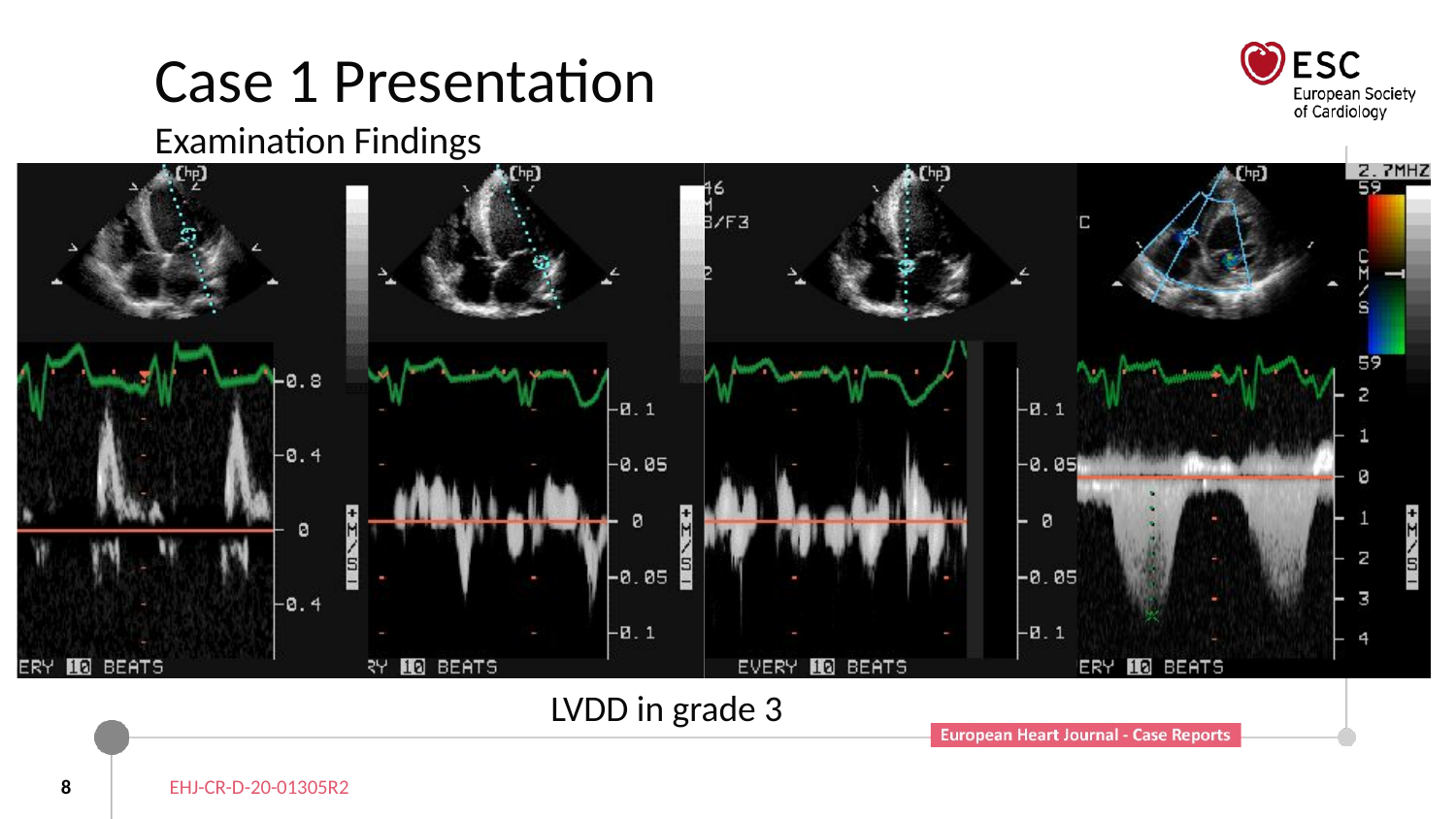

# Case 1 Presentation Examination Findings
LVDD in grade 3
8
EHJ-CR-D-20-01305R2

## Slide 9
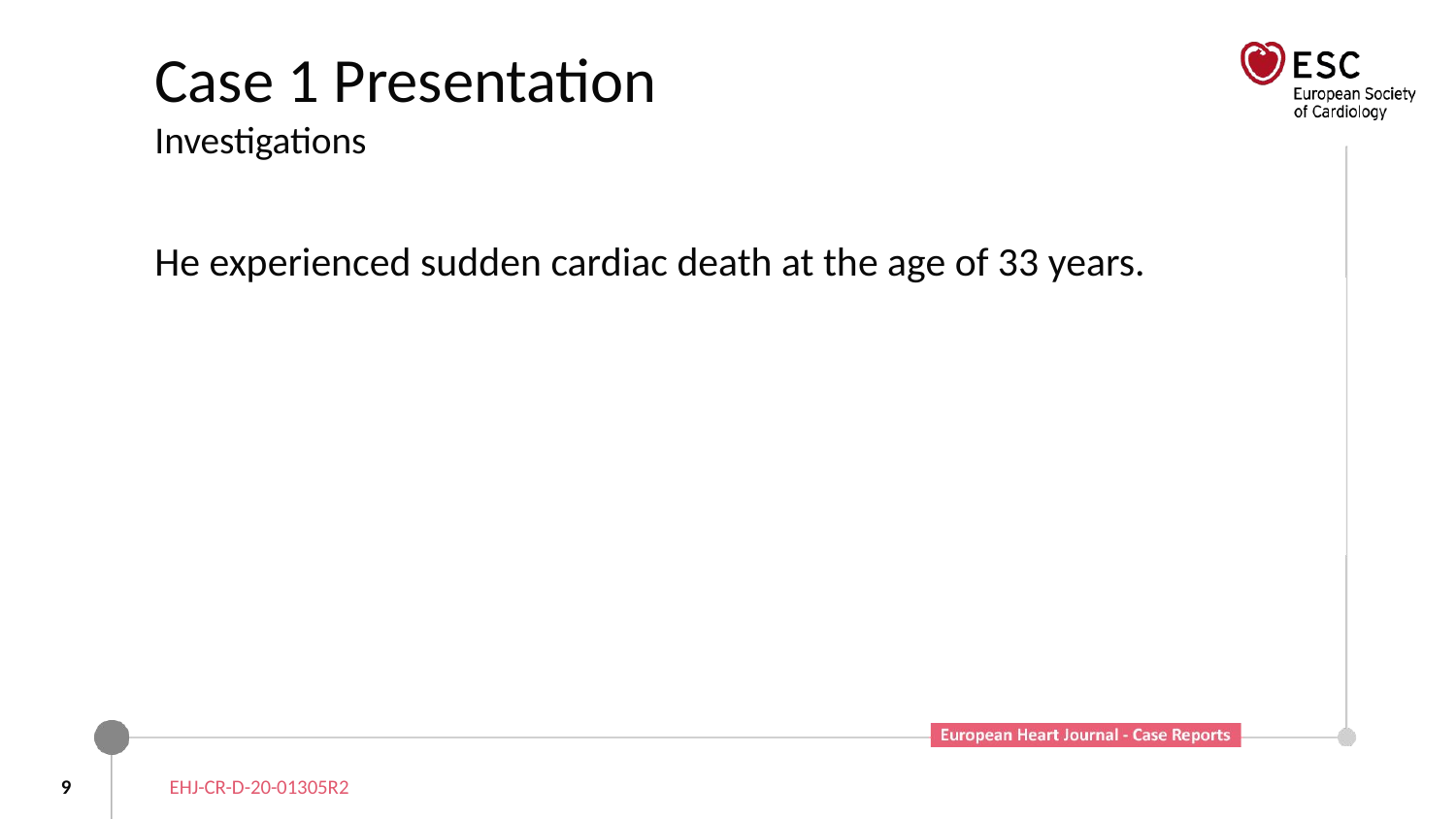

# Case 1 Presentation Investigations
He experienced sudden cardiac death at the age of 33 years.
9
EHJ-CR-D-20-01305R2

## Slide 10
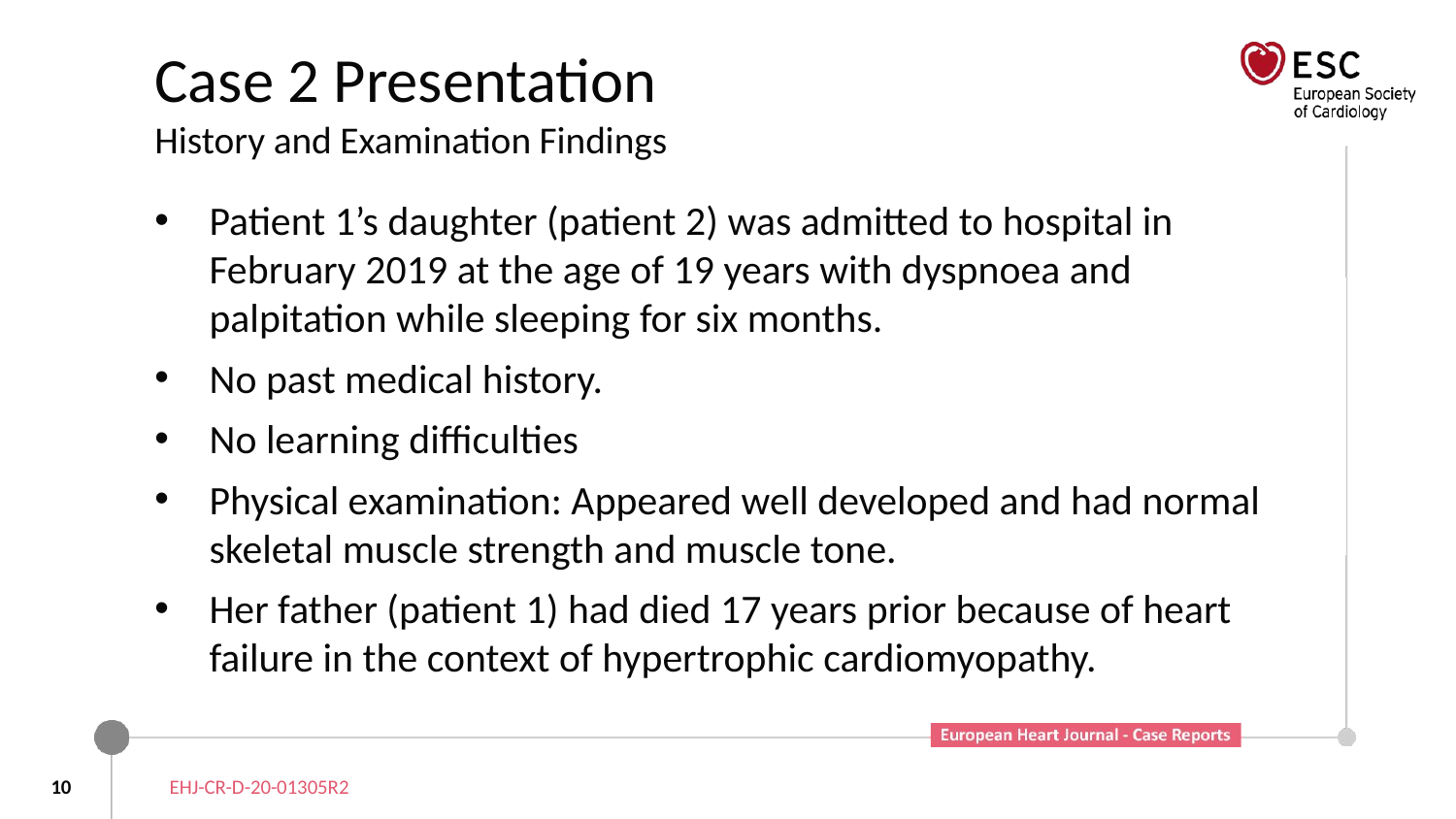

# Case 2 Presentation History and Examination Findings
Patient 1’s daughter (patient 2) was admitted to hospital in February 2019 at the age of 19 years with dyspnoea and palpitation while sleeping for six months.
No past medical history.
No learning difficulties
Physical examination: Appeared well developed and had normal skeletal muscle strength and muscle tone.
Her father (patient 1) had died 17 years prior because of heart failure in the context of hypertrophic cardiomyopathy.
10
EHJ-CR-D-20-01305R2

## Slide 11
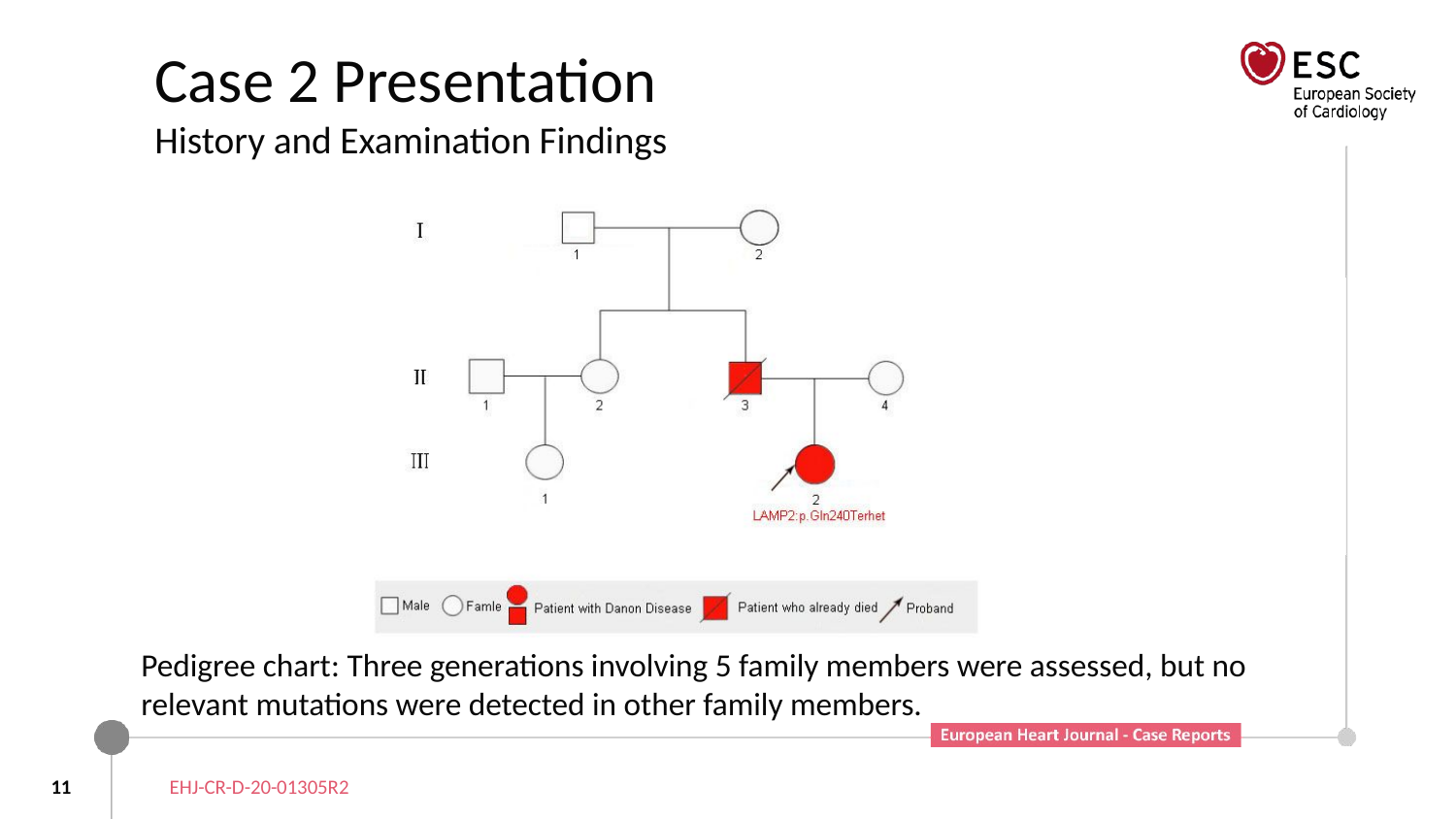

# Case 2 Presentation History and Examination Findings
Pedigree chart: Three generations involving 5 family members were assessed, but no relevant mutations were detected in other family members.
11
EHJ-CR-D-20-01305R2

## Slide 12
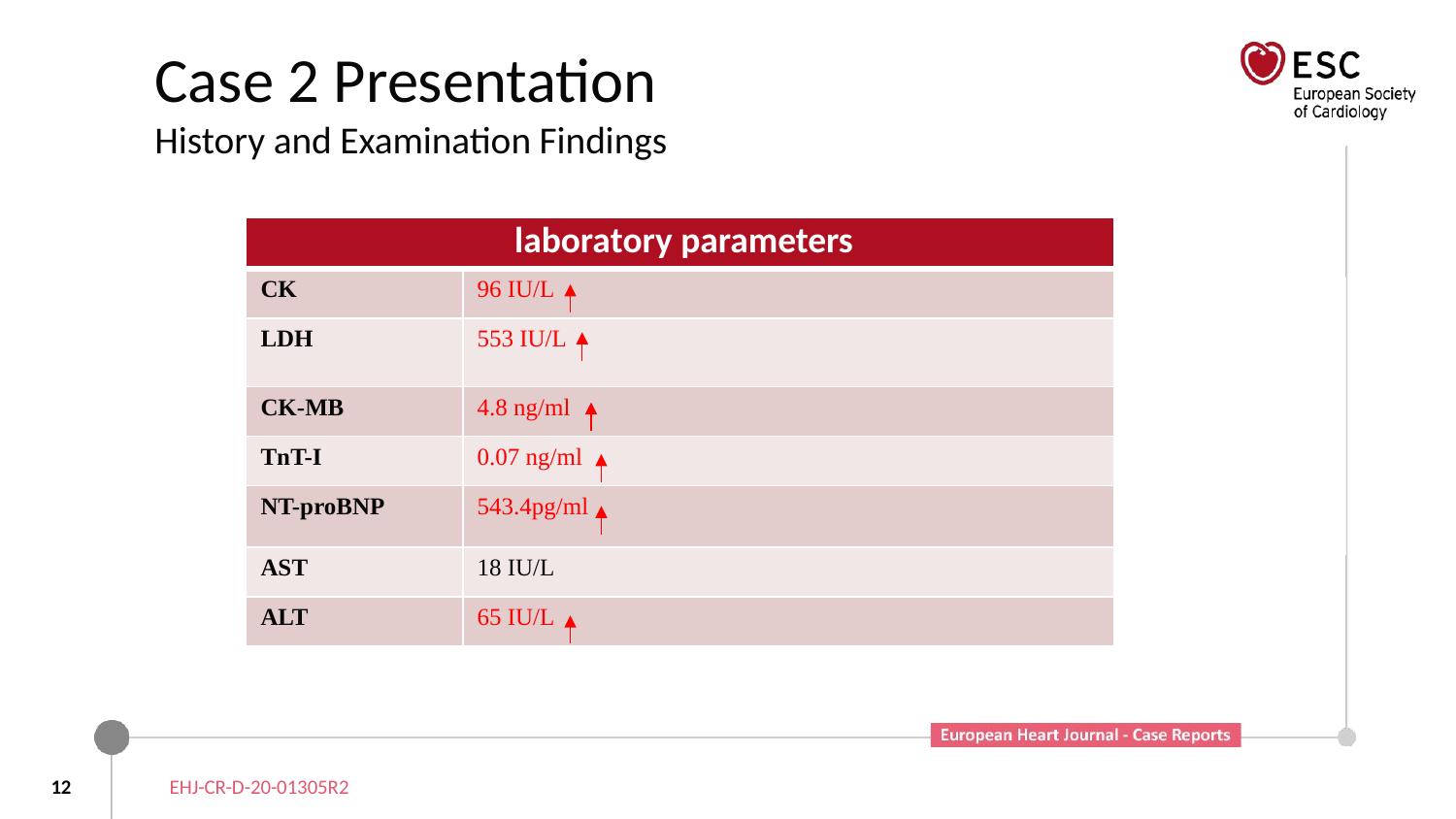

# Case 2 Presentation History and Examination Findings
| laboratory parameters | |
| --- | --- |
| CK | 96 IU/L |
| LDH | 553 IU/L |
| CK-MB | 4.8 ng/ml |
| TnT-I | 0.07 ng/ml |
| NT-proBNP | 543.4pg/ml |
| AST | 18 IU/L |
| ALT | 65 IU/L |
12
EHJ-CR-D-20-01305R2

## Slide 13
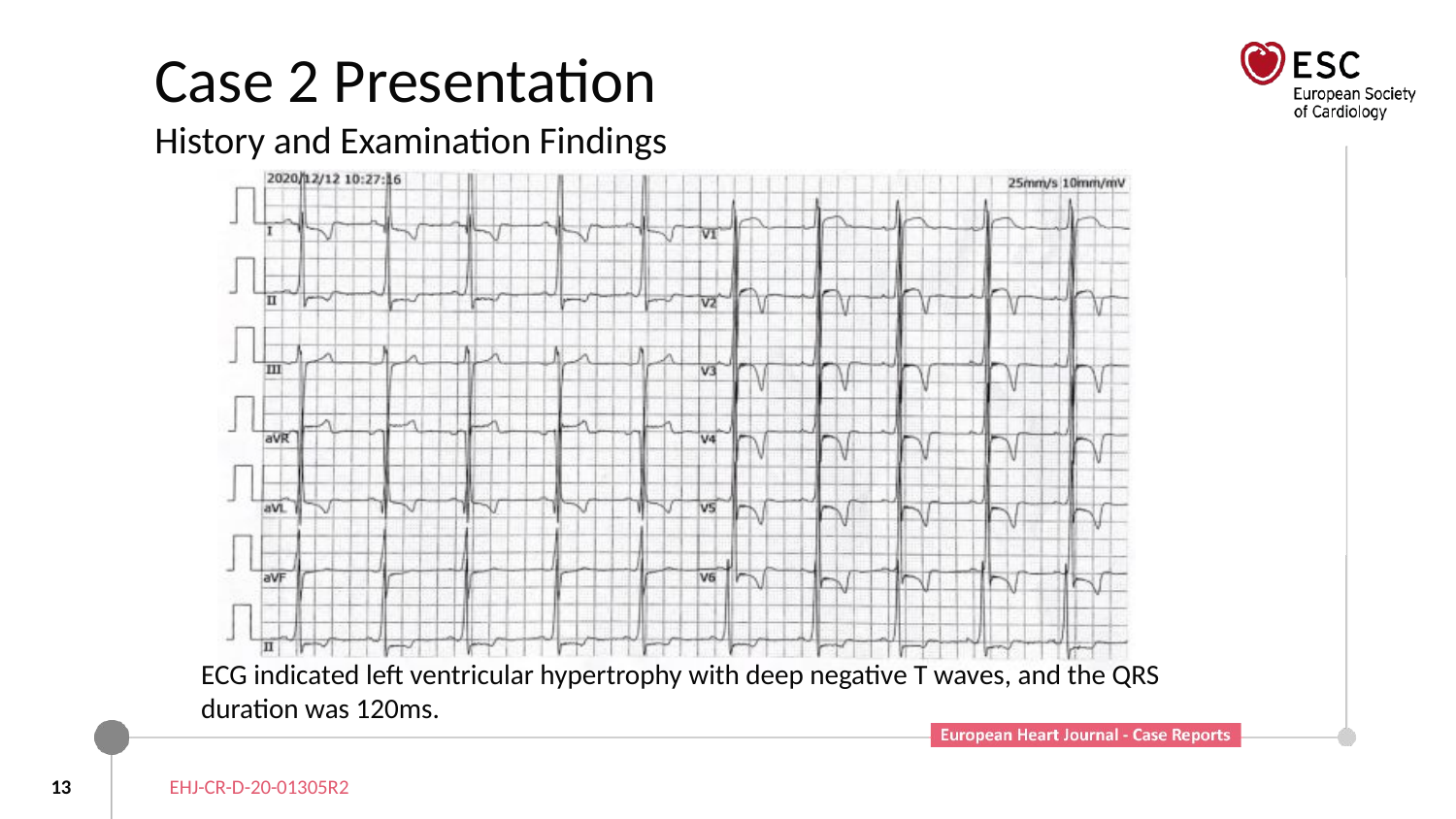

# Case 2 Presentation History and Examination Findings
ECG indicated left ventricular hypertrophy with deep negative T waves, and the QRS duration was 120ms.
13
EHJ-CR-D-20-01305R2

## Slide 14
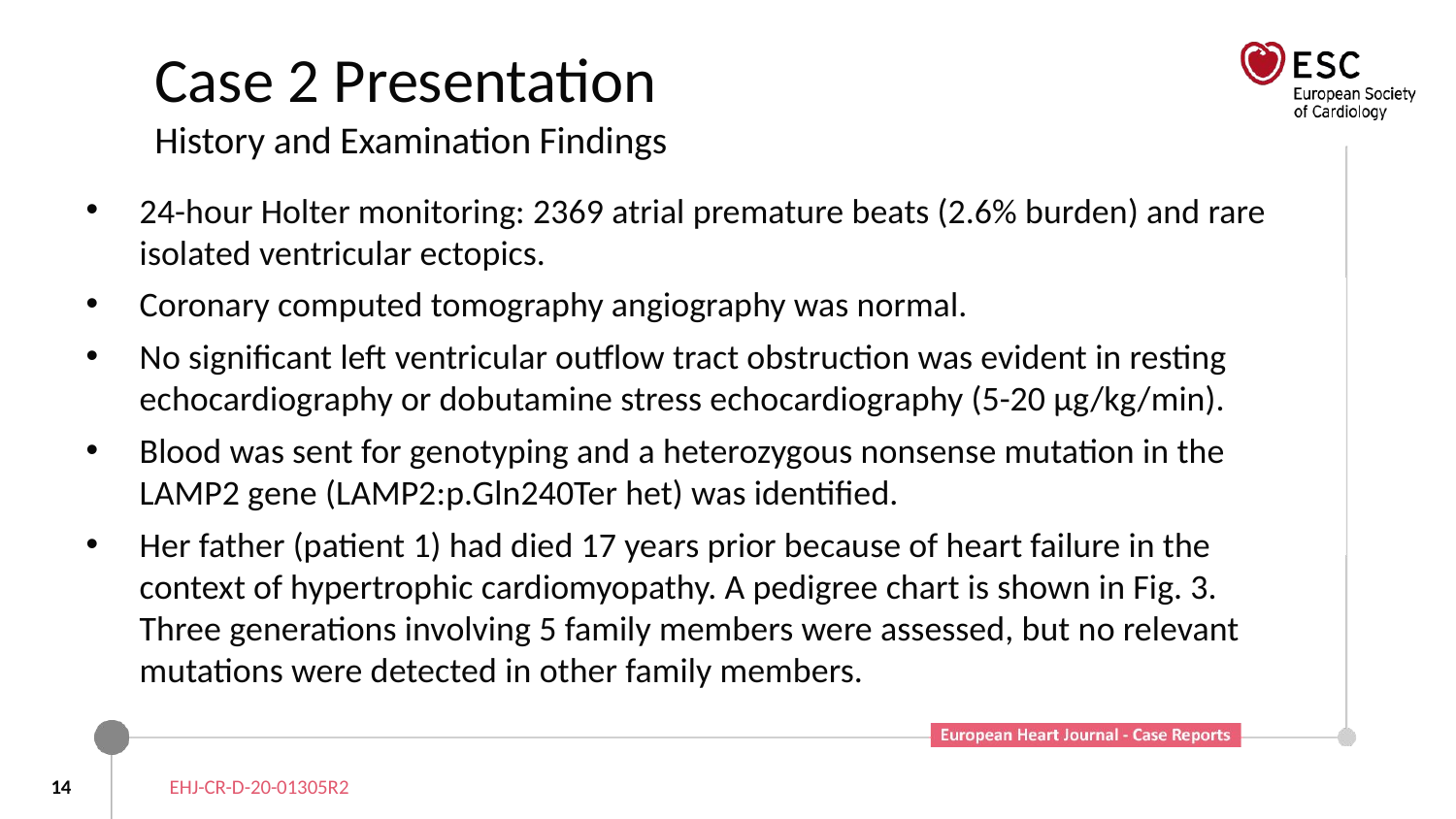

# Case 2 Presentation History and Examination Findings
24-hour Holter monitoring: 2369 atrial premature beats (2.6% burden) and rare isolated ventricular ectopics.
Coronary computed tomography angiography was normal.
No significant left ventricular outflow tract obstruction was evident in resting echocardiography or dobutamine stress echocardiography (5-20 µg/kg/min).
Blood was sent for genotyping and a heterozygous nonsense mutation in the LAMP2 gene (LAMP2:p.Gln240Ter het) was identified.
Her father (patient 1) had died 17 years prior because of heart failure in the context of hypertrophic cardiomyopathy. A pedigree chart is shown in Fig. 3. Three generations involving 5 family members were assessed, but no relevant mutations were detected in other family members.
14
EHJ-CR-D-20-01305R2

## Slide 15
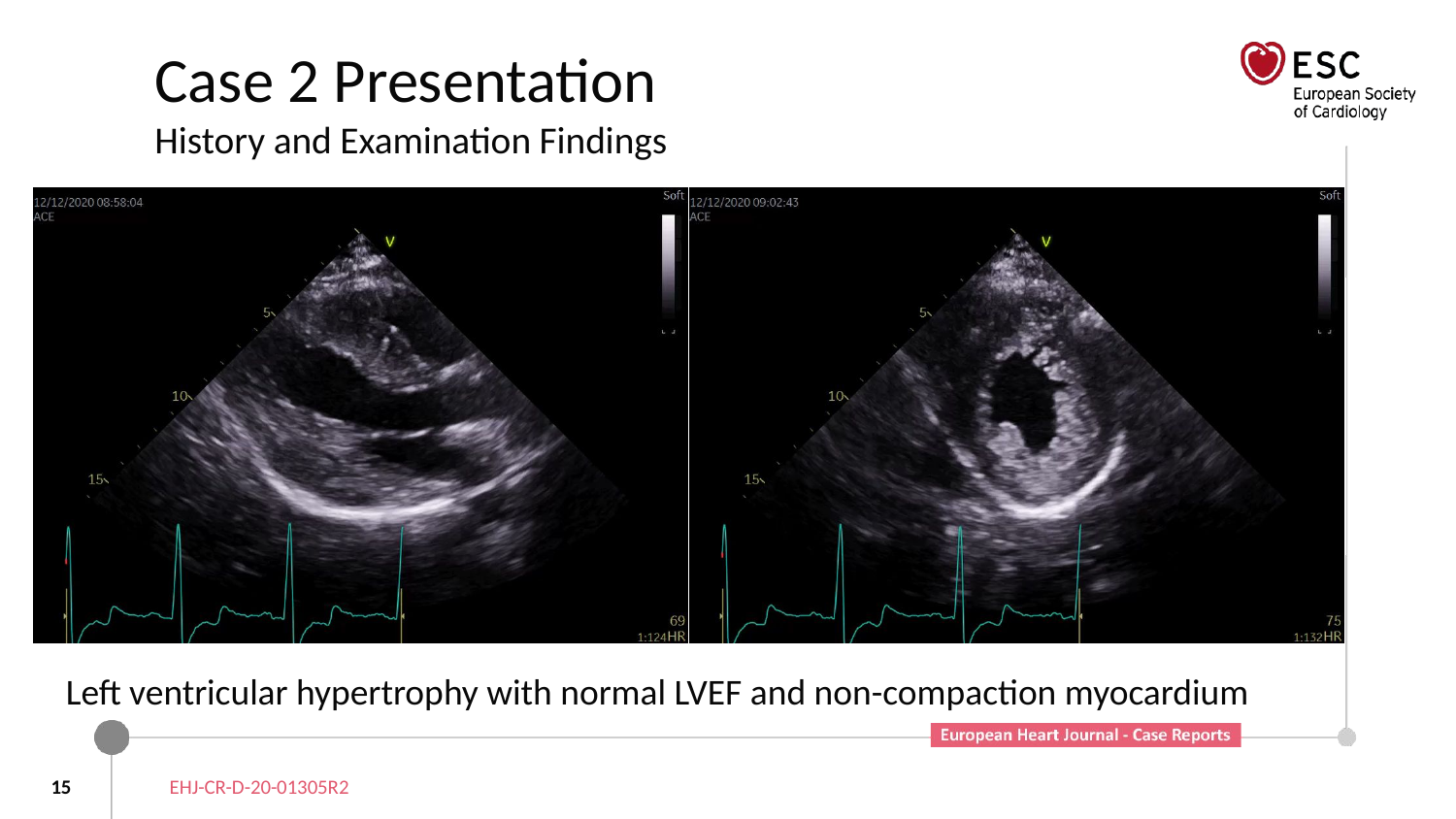

# Case 2 Presentation History and Examination Findings
Left ventricular hypertrophy with normal LVEF and non-compaction myocardium
15
EHJ-CR-D-20-01305R2

## Slide 16
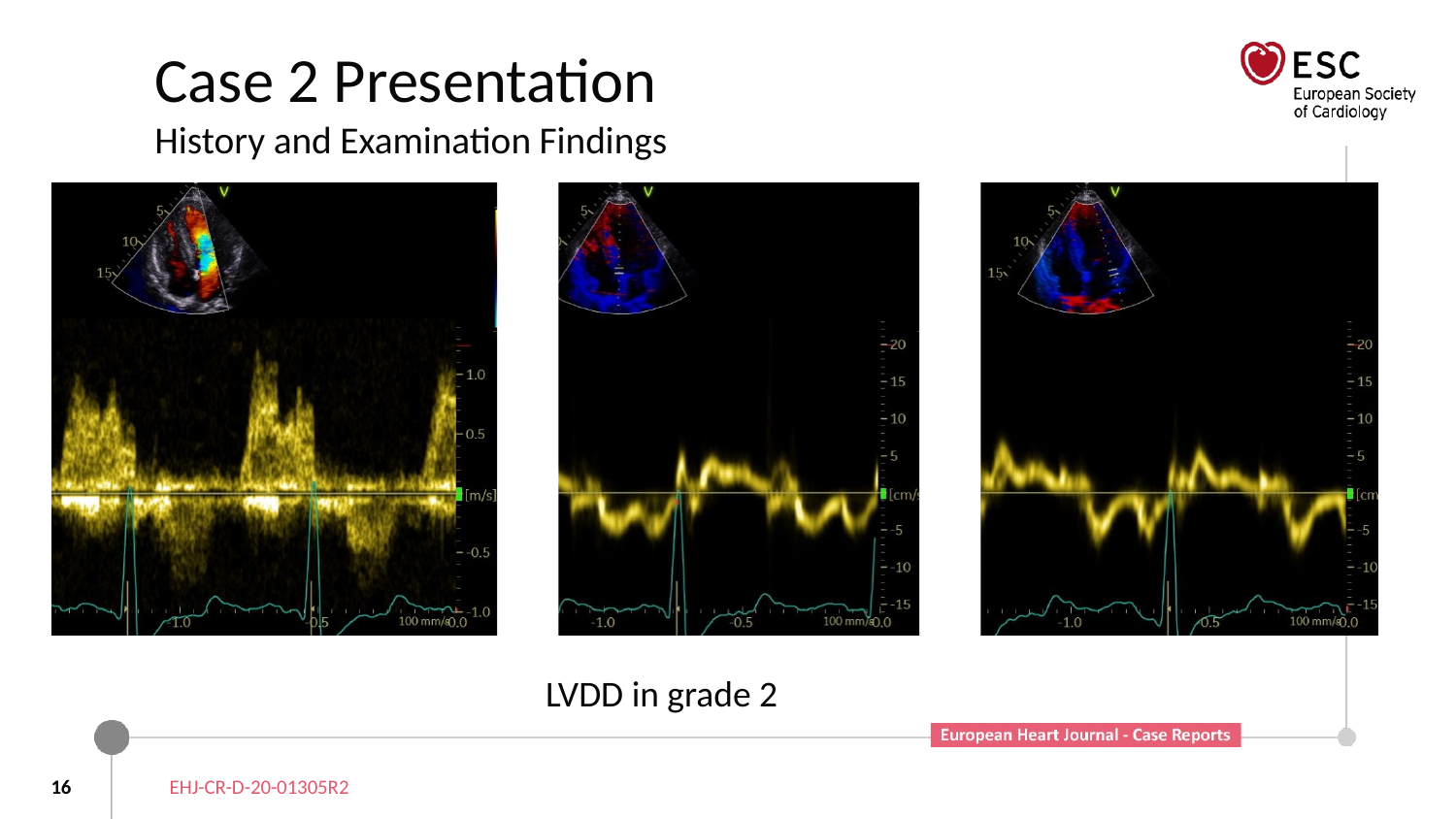

# Case 2 Presentation History and Examination Findings
LVDD in grade 2
16
EHJ-CR-D-20-01305R2

## Slide 17
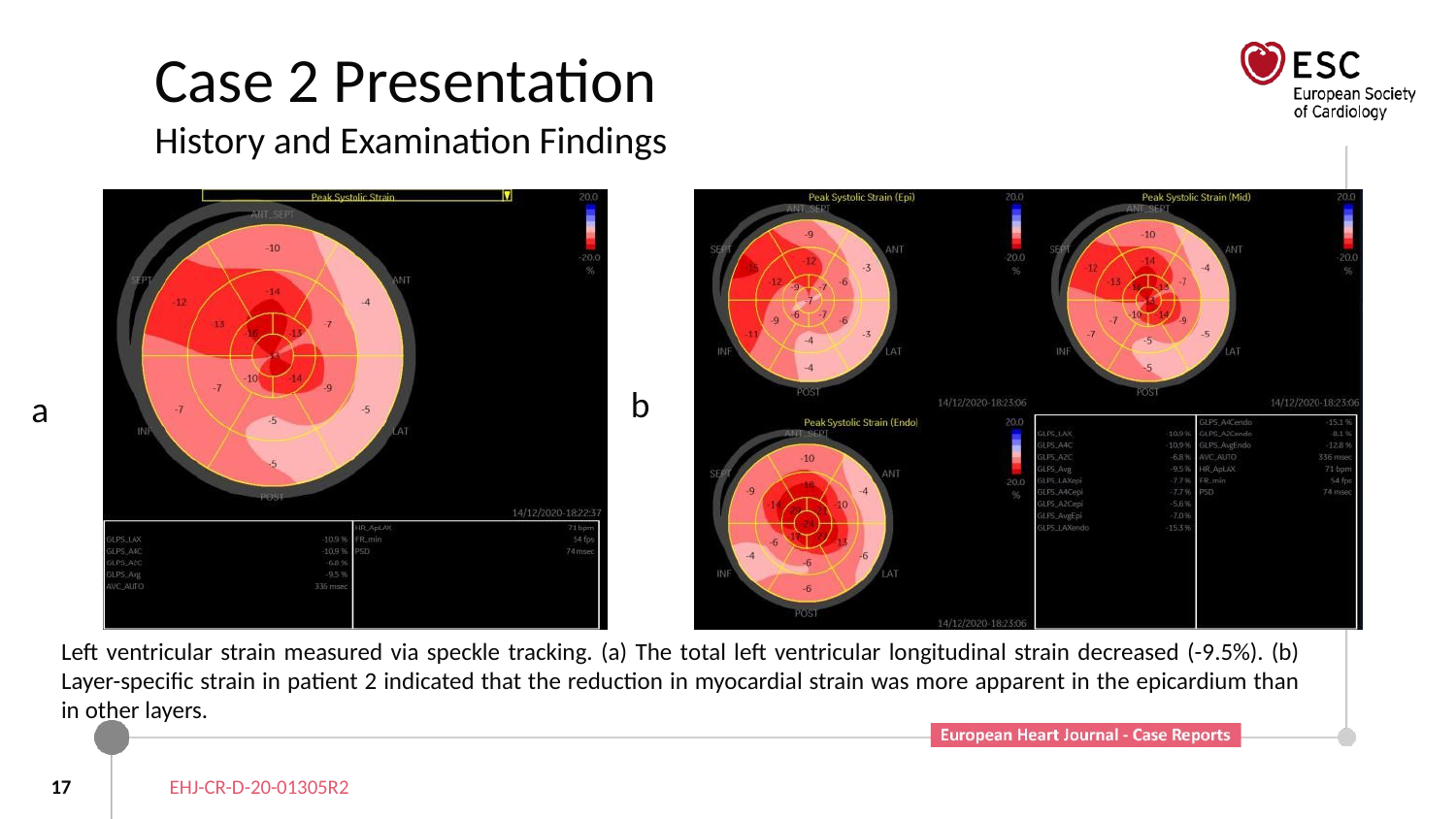

# Case 2 Presentation History and Examination Findings
b
a
Left ventricular strain measured via speckle tracking. (a) The total left ventricular longitudinal strain decreased (-9.5%). (b) Layer-specific strain in patient 2 indicated that the reduction in myocardial strain was more apparent in the epicardium than in other layers.
17
EHJ-CR-D-20-01305R2

## Slide 18
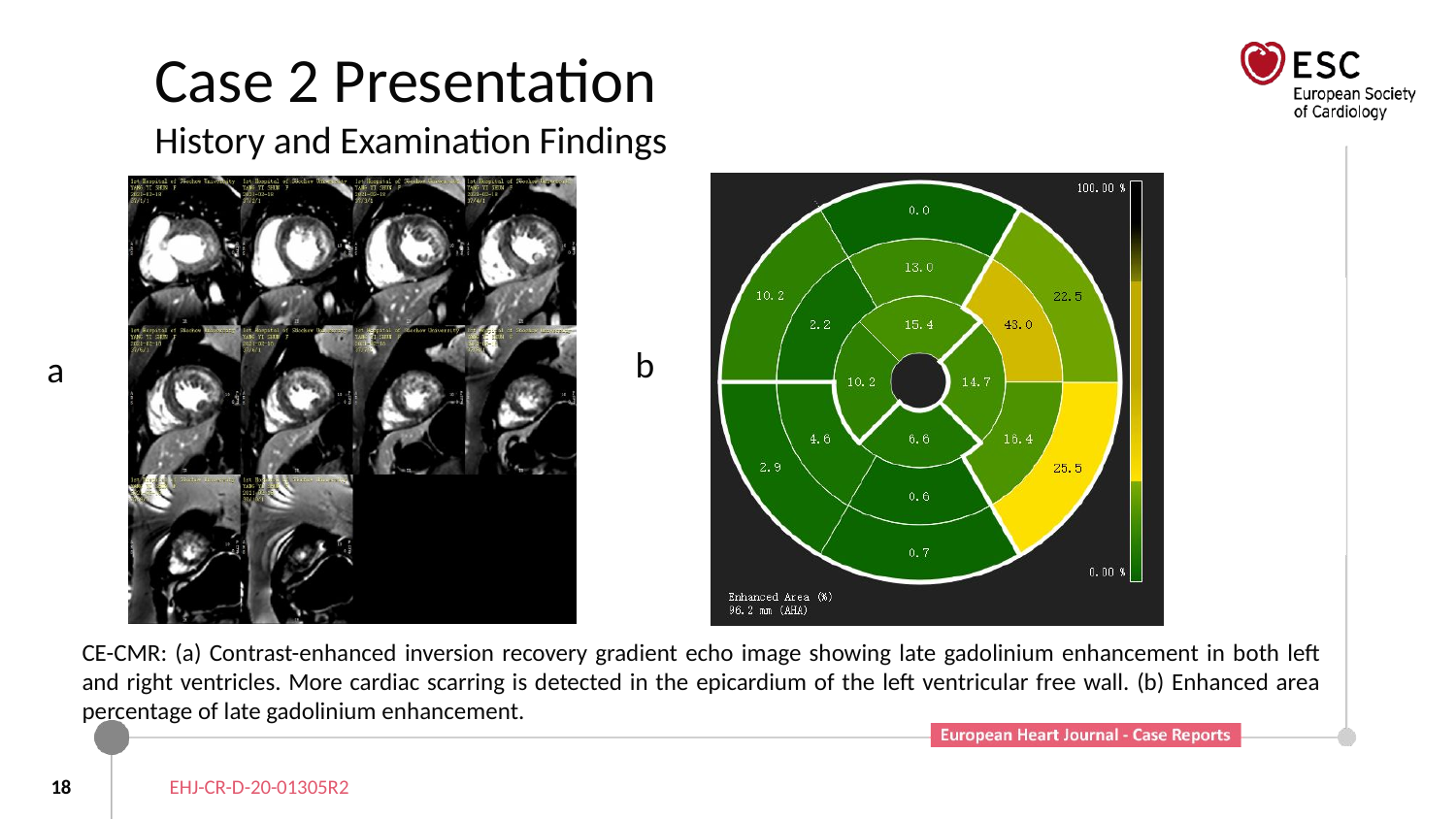

# Case 2 Presentation History and Examination Findings
b
a
CE-CMR: (a) Contrast-enhanced inversion recovery gradient echo image showing late gadolinium enhancement in both left and right ventricles. More cardiac scarring is detected in the epicardium of the left ventricular free wall. (b) Enhanced area percentage of late gadolinium enhancement.
18
EHJ-CR-D-20-01305R2

## Slide 19
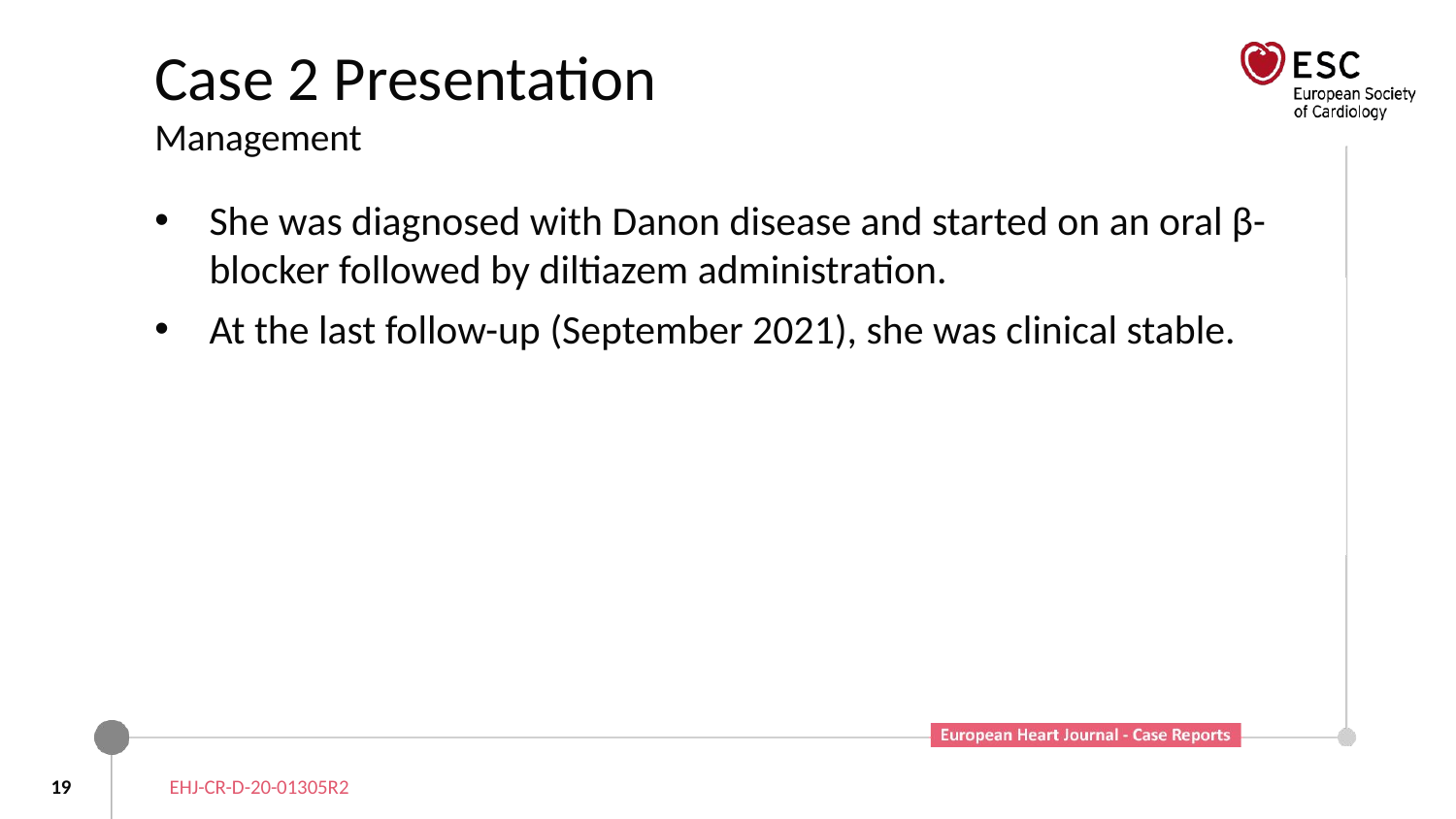

# Case 2 Presentation Management
She was diagnosed with Danon disease and started on an oral β-blocker followed by diltiazem administration.
At the last follow-up (September 2021), she was clinical stable.
19
EHJ-CR-D-20-01305R2

## Slide 20
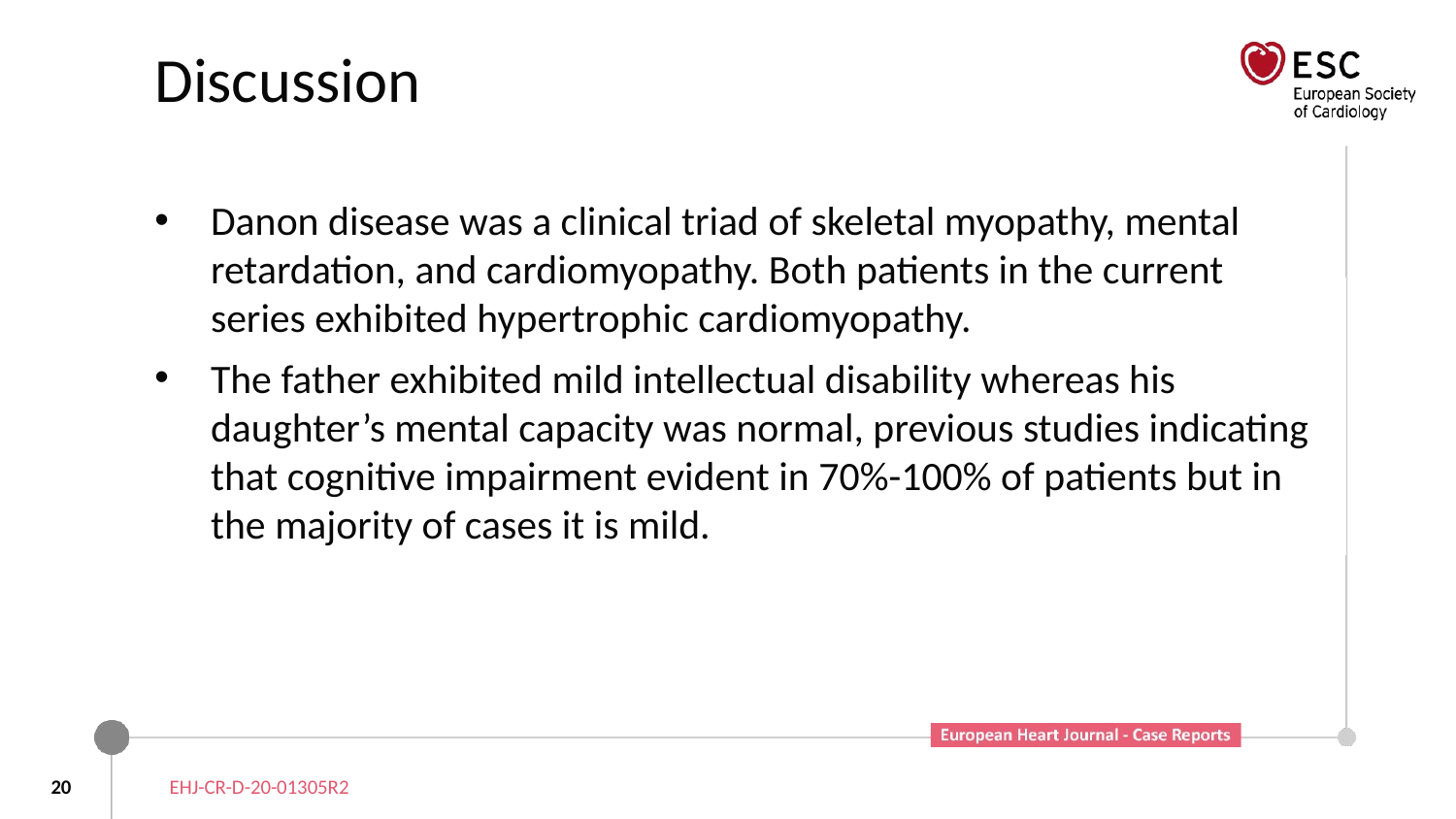

# Discussion
Danon disease was a clinical triad of skeletal myopathy, mental retardation, and cardiomyopathy. Both patients in the current series exhibited hypertrophic cardiomyopathy.
The father exhibited mild intellectual disability whereas his daughter’s mental capacity was normal, previous studies indicating that cognitive impairment evident in 70%-100% of patients but in the majority of cases it is mild.
20
EHJ-CR-D-20-01305R2

## Slide 21
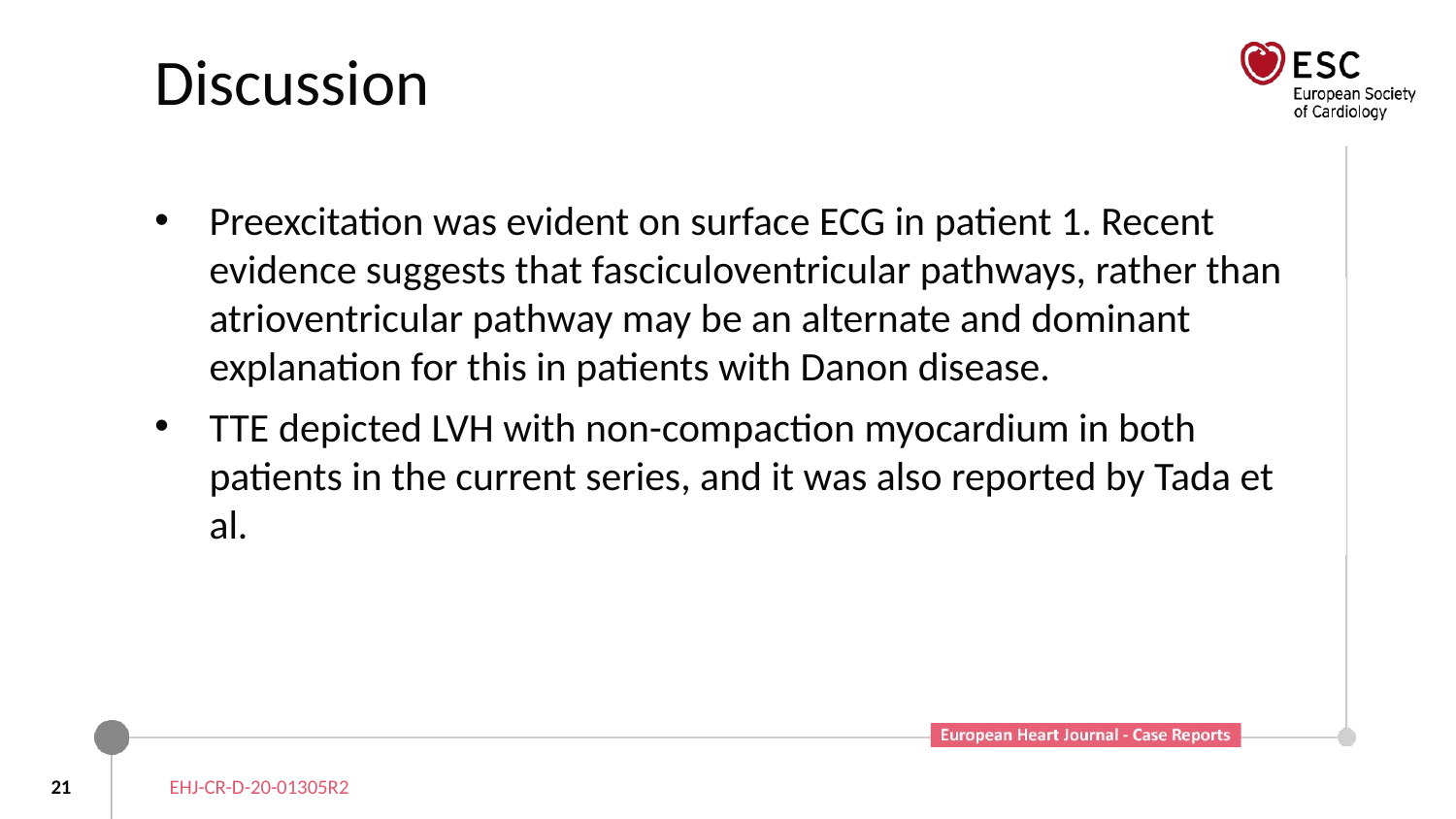

# Discussion
Preexcitation was evident on surface ECG in patient 1. Recent evidence suggests that fasciculoventricular pathways, rather than atrioventricular pathway may be an alternate and dominant explanation for this in patients with Danon disease.
TTE depicted LVH with non-compaction myocardium in both patients in the current series, and it was also reported by Tada et al.
21
EHJ-CR-D-20-01305R2

## Slide 22
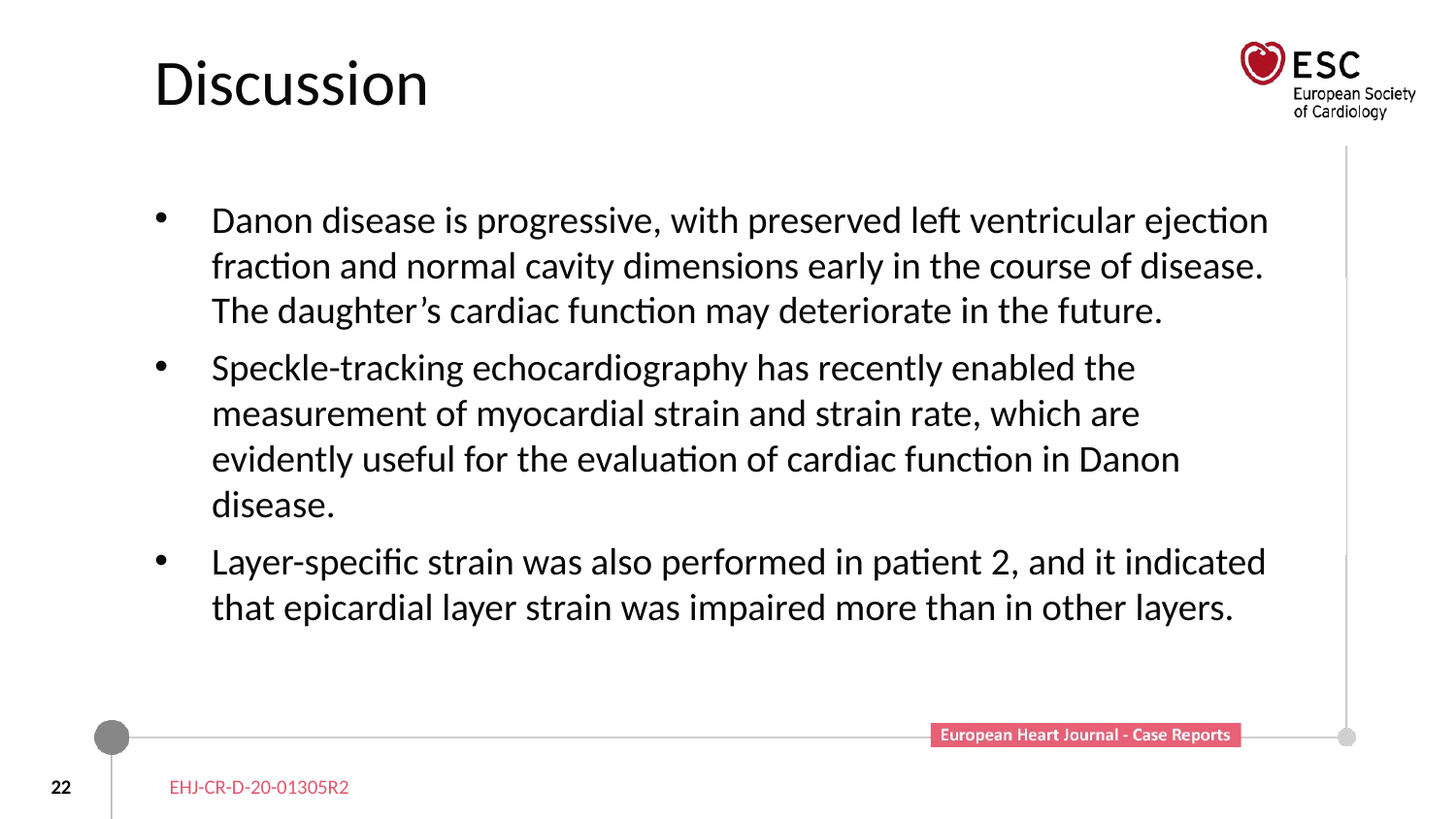

# Discussion
Danon disease is progressive, with preserved left ventricular ejection fraction and normal cavity dimensions early in the course of disease. The daughter’s cardiac function may deteriorate in the future.
Speckle-tracking echocardiography has recently enabled the measurement of myocardial strain and strain rate, which are evidently useful for the evaluation of cardiac function in Danon disease.
Layer-specific strain was also performed in patient 2, and it indicated that epicardial layer strain was impaired more than in other layers.
22
EHJ-CR-D-20-01305R2

## Slide 23
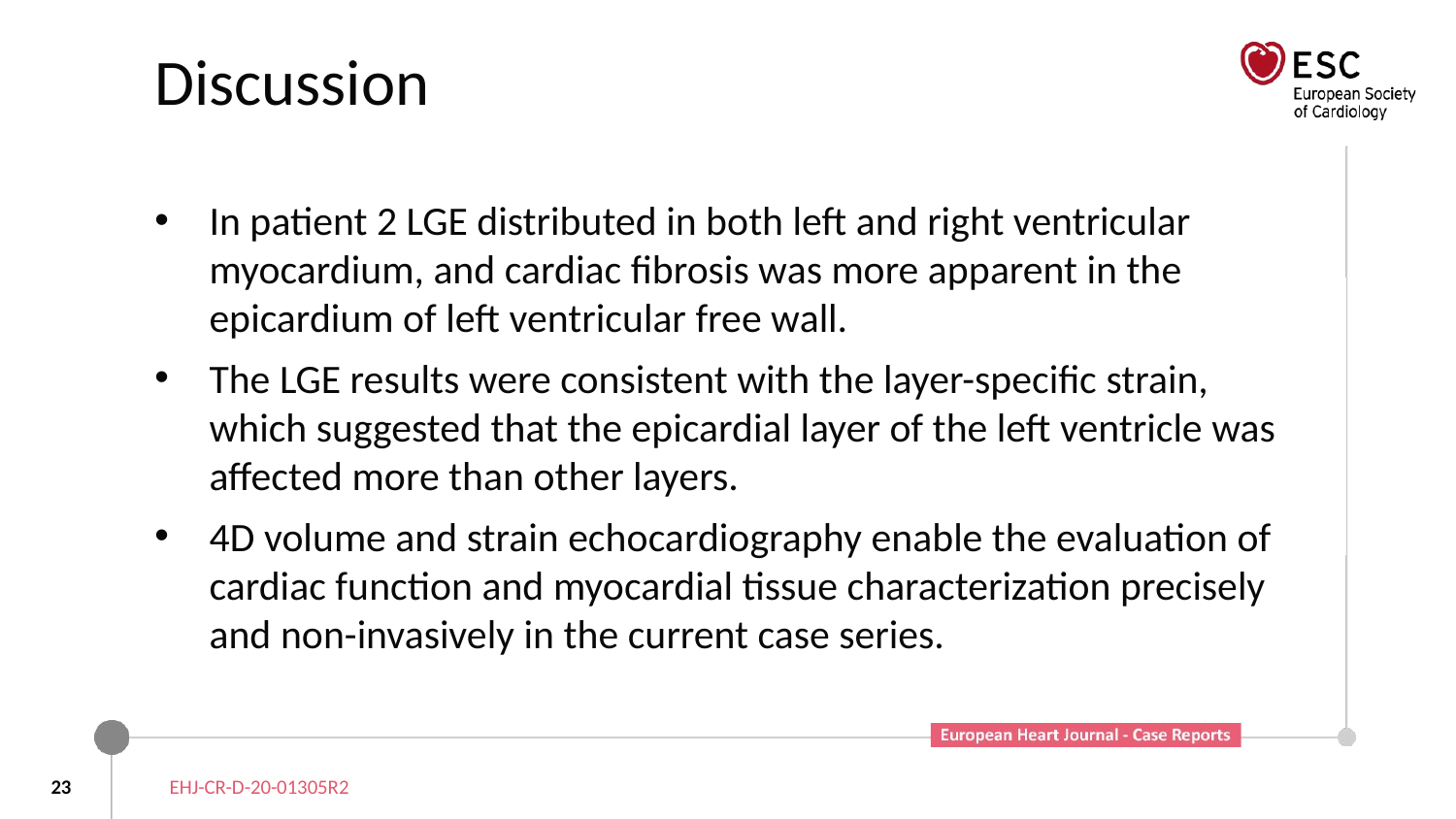

# Discussion
In patient 2 LGE distributed in both left and right ventricular myocardium, and cardiac fibrosis was more apparent in the epicardium of left ventricular free wall.
The LGE results were consistent with the layer-specific strain, which suggested that the epicardial layer of the left ventricle was affected more than other layers.
4D volume and strain echocardiography enable the evaluation of cardiac function and myocardial tissue characterization precisely and non-invasively in the current case series.
23
EHJ-CR-D-20-01305R2

## Slide 24
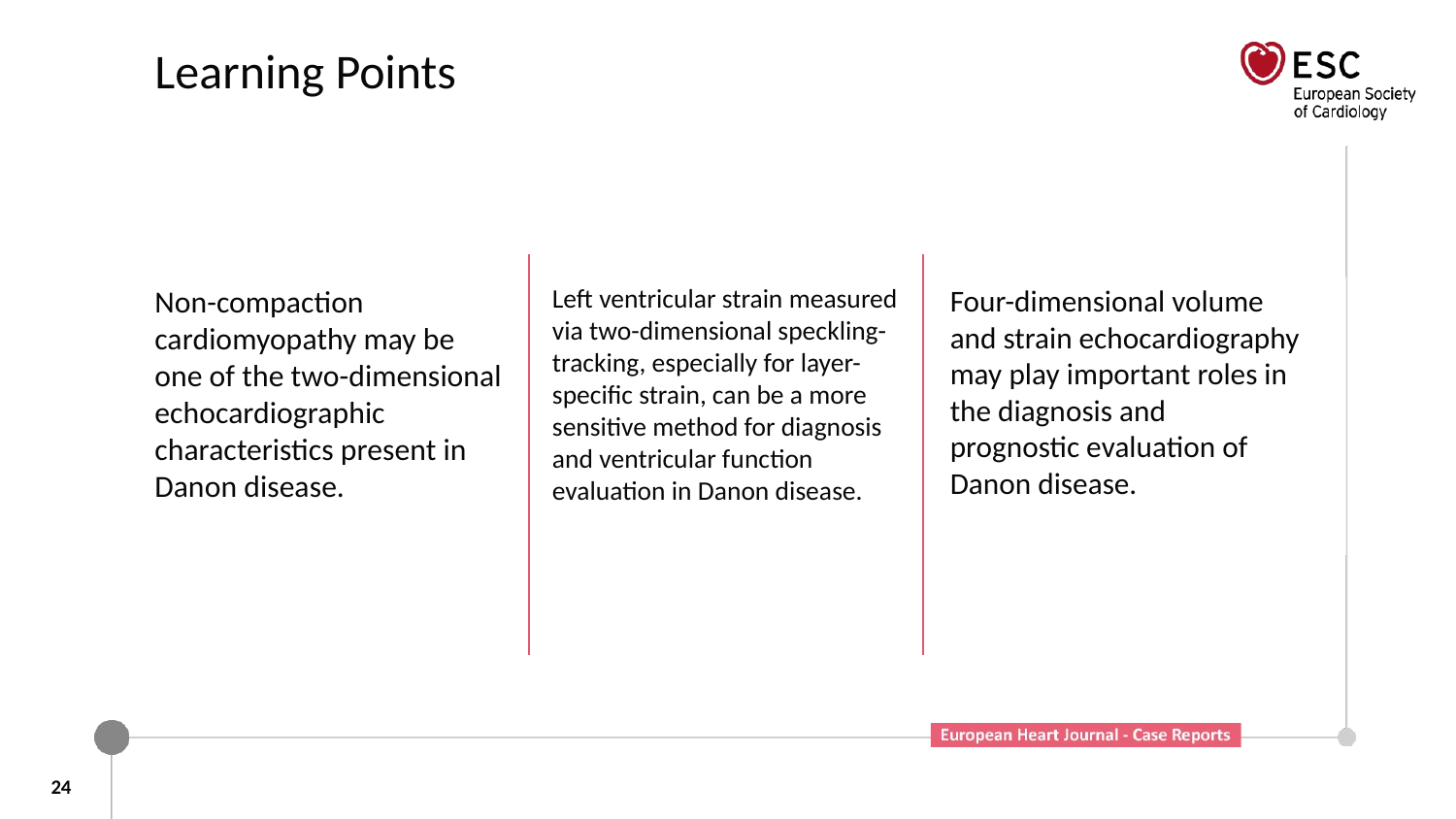

# Learning Points
Non-compaction cardiomyopathy may be one of the two-dimensional echocardiographic characteristics present in Danon disease.
Left ventricular strain measured via two-dimensional speckling-tracking, especially for layer-specific strain, can be a more sensitive method for diagnosis and ventricular function evaluation in Danon disease.
Four-dimensional volume and strain echocardiography may play important roles in the diagnosis and prognostic evaluation of Danon disease.
24

## Slide 25
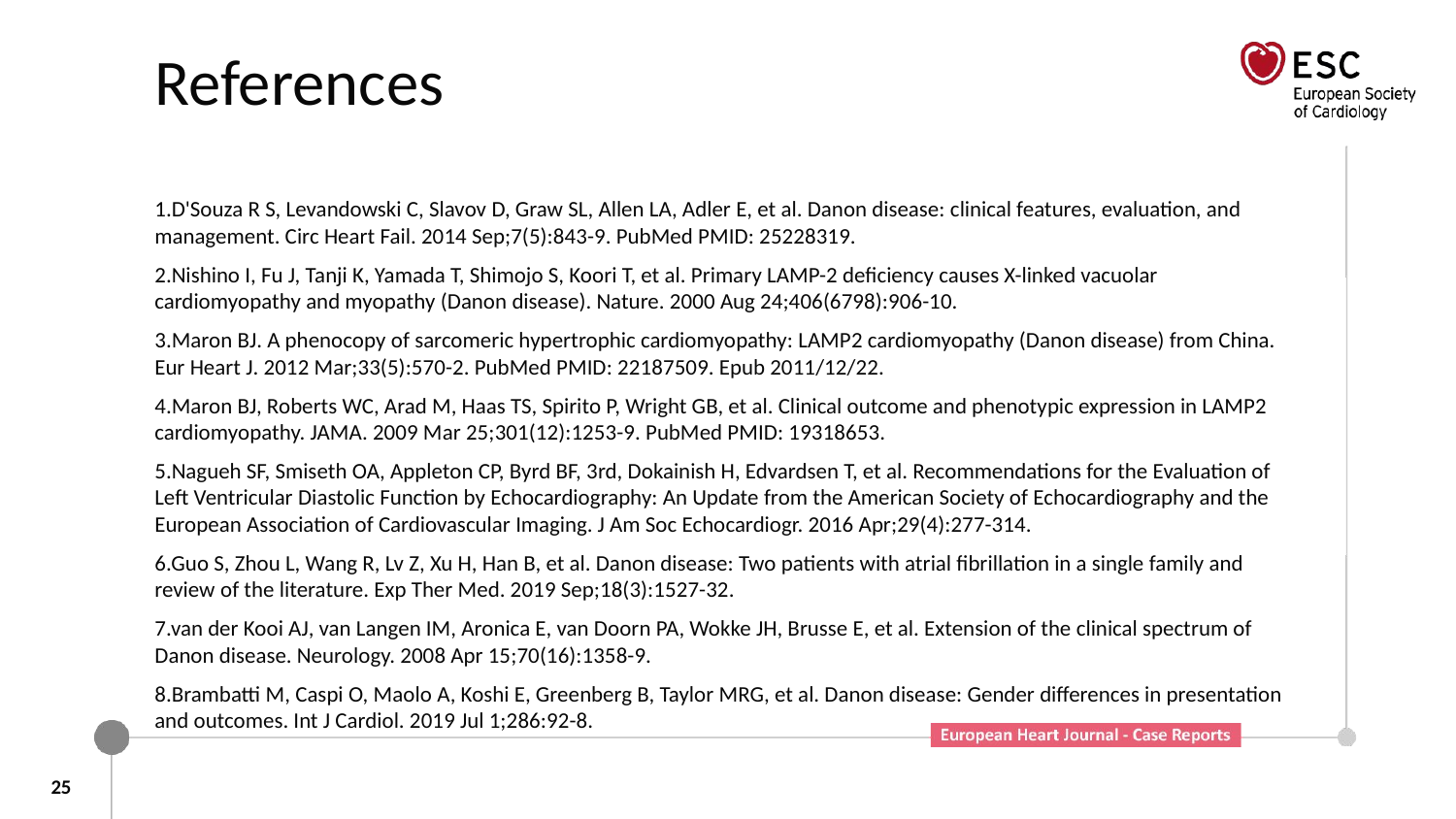

# References
1.D'Souza R S, Levandowski C, Slavov D, Graw SL, Allen LA, Adler E, et al. Danon disease: clinical features, evaluation, and management. Circ Heart Fail. 2014 Sep;7(5):843-9. PubMed PMID: 25228319.
2.Nishino I, Fu J, Tanji K, Yamada T, Shimojo S, Koori T, et al. Primary LAMP-2 deficiency causes X-linked vacuolar cardiomyopathy and myopathy (Danon disease). Nature. 2000 Aug 24;406(6798):906-10.
3.Maron BJ. A phenocopy of sarcomeric hypertrophic cardiomyopathy: LAMP2 cardiomyopathy (Danon disease) from China. Eur Heart J. 2012 Mar;33(5):570-2. PubMed PMID: 22187509. Epub 2011/12/22.
4.Maron BJ, Roberts WC, Arad M, Haas TS, Spirito P, Wright GB, et al. Clinical outcome and phenotypic expression in LAMP2 cardiomyopathy. JAMA. 2009 Mar 25;301(12):1253-9. PubMed PMID: 19318653.
5.Nagueh SF, Smiseth OA, Appleton CP, Byrd BF, 3rd, Dokainish H, Edvardsen T, et al. Recommendations for the Evaluation of Left Ventricular Diastolic Function by Echocardiography: An Update from the American Society of Echocardiography and the European Association of Cardiovascular Imaging. J Am Soc Echocardiogr. 2016 Apr;29(4):277-314.
6.Guo S, Zhou L, Wang R, Lv Z, Xu H, Han B, et al. Danon disease: Two patients with atrial fibrillation in a single family and review of the literature. Exp Ther Med. 2019 Sep;18(3):1527-32.
7.van der Kooi AJ, van Langen IM, Aronica E, van Doorn PA, Wokke JH, Brusse E, et al. Extension of the clinical spectrum of Danon disease. Neurology. 2008 Apr 15;70(16):1358-9.
8.Brambatti M, Caspi O, Maolo A, Koshi E, Greenberg B, Taylor MRG, et al. Danon disease: Gender differences in presentation and outcomes. Int J Cardiol. 2019 Jul 1;286:92-8.
25

## Slide 26
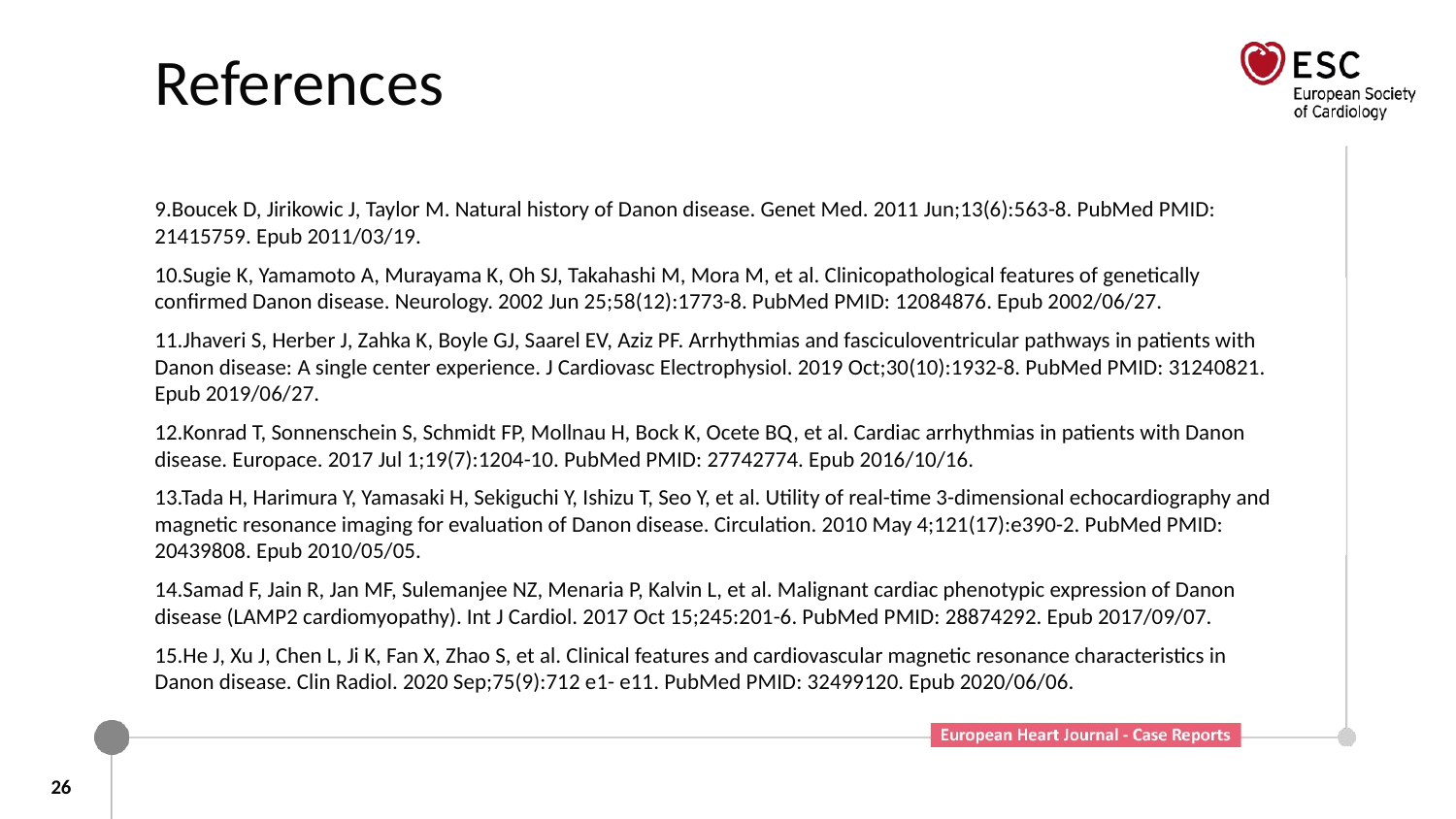

# References
9.Boucek D, Jirikowic J, Taylor M. Natural history of Danon disease. Genet Med. 2011 Jun;13(6):563-8. PubMed PMID: 21415759. Epub 2011/03/19.
10.Sugie K, Yamamoto A, Murayama K, Oh SJ, Takahashi M, Mora M, et al. Clinicopathological features of genetically confirmed Danon disease. Neurology. 2002 Jun 25;58(12):1773-8. PubMed PMID: 12084876. Epub 2002/06/27.
11.Jhaveri S, Herber J, Zahka K, Boyle GJ, Saarel EV, Aziz PF. Arrhythmias and fasciculoventricular pathways in patients with Danon disease: A single center experience. J Cardiovasc Electrophysiol. 2019 Oct;30(10):1932-8. PubMed PMID: 31240821. Epub 2019/06/27.
12.Konrad T, Sonnenschein S, Schmidt FP, Mollnau H, Bock K, Ocete BQ, et al. Cardiac arrhythmias in patients with Danon disease. Europace. 2017 Jul 1;19(7):1204-10. PubMed PMID: 27742774. Epub 2016/10/16.
13.Tada H, Harimura Y, Yamasaki H, Sekiguchi Y, Ishizu T, Seo Y, et al. Utility of real-time 3-dimensional echocardiography and magnetic resonance imaging for evaluation of Danon disease. Circulation. 2010 May 4;121(17):e390-2. PubMed PMID: 20439808. Epub 2010/05/05.
14.Samad F, Jain R, Jan MF, Sulemanjee NZ, Menaria P, Kalvin L, et al. Malignant cardiac phenotypic expression of Danon disease (LAMP2 cardiomyopathy). Int J Cardiol. 2017 Oct 15;245:201-6. PubMed PMID: 28874292. Epub 2017/09/07.
15.He J, Xu J, Chen L, Ji K, Fan X, Zhao S, et al. Clinical features and cardiovascular magnetic resonance characteristics in Danon disease. Clin Radiol. 2020 Sep;75(9):712 e1- e11. PubMed PMID: 32499120. Epub 2020/06/06.
26
